# Laser‐Induced Graphene from Commercial Inks and Dyes

**DOI:** 10.1002/advs.202412167

**Published:** 2025-02-14

**Authors:** Alexander Dallinger, Rachel Camerini, Paola Parlanti, Mauro Gemmi, Sreenadh Thaikkattu Sankaran, Marina Galliani, Birgit Kunert, Rodorico Giorgi, Francesco Greco

**Affiliations:** ^1^ Institute of Solid State Physics NAWI Graz Graz University of Technology Graz 8010 Austria; ^2^ Center for Colloid and Surface Science – CSGI and Department of Chemistry University of Florence Via della Lastruccia 3 Sesto Fiorentino 50019 Italy; ^3^ Center for Materials Interfaces Electron Crystallography Istituto Italiano di Tecnologia Viale R. Piaggio 34 Pontedera 56025 Italy; ^4^ The Biorobotics Institute and Dept. of Excellence in Robotics & AI Scuola Superiore Sant'Anna Viale R. Piaggio 34 Pontedera 56025 Italy; ^5^ Interdisciplinary Center on Sustainability and Climate Scuola Superiore Sant'Anna Piazza Martiri della Libertà 33 Pisa 56127 Italy

**Keywords:** dye, Eosin Y, freestanding, ink, laser‐induced graphene, pyrolysis, transfer

## Abstract

Laser‐induced graphene (LIG) has been so far obtained from polymer precursors and proposed for numerous applications, including various types of sensors and energy storage solutions. This study examines a radically different class of new precursors for LIG, distinct from polymers: inks and dyes. The identification of specific organic dyes present in commercial markers demonstrates that the aromatic structure, in conjunction with high thermal stability (residual weight > 20% at 800°C), are key factors for laser‐induced pyrolysis. Eosin Y is identified as an excellent LIG precursor, comparable with well‐known polyimide. The unique properties of dyes allow for dispersion in various media, such as acrylic binder. A dye concentration of 0.75 mol L^−1^ in acrylic binder results in a conductivity of 34 ± 20 S cm^−1^ for LIG. The composition and microstructure of LIG from dyes are thoroughly characterized, revealing peculiar features. A versatile “Paint & Scribe” methodology is introduced, enabling to integrate LIG tracks onto any wettable surface, and in particular onto printed and flexible electronics. A process for obtaining freestanding and transferrable LIG is demonstrated by dissolving acrylic paint in acetone and floating LIG in water. This advancement offers novel avenues for diverse applications that necessitate a transfer process of LIG.

## Graphical abstract

Dyes are identified as a new class of precursor for Laser‐Induced Graphene (LIG). The conversion via laser‐induced pyrolysis and resulting LIG are systematically characterized. A new methodology called “Paint & Scribe” is introduced which allows the functionalization of any wettable surface with LIG and an integration with printed and flexible electronics.

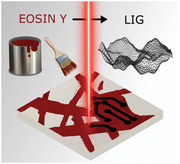

## 1 Introduction

The use of Direct Laser Writing (DLW) for manufacturing and laser‐based processing is increasing due to the availability of low‐cost lasers. One distinguishes between additive, subtractive, and transformative DLW. The latter is a special case where a precursor material is chemically or structurally changed by the laser irradiation as in the case of laser‐induced pyrolysis.^[^ ^1^ ^]^ The discovery of laser‐induced pyrolysis of polymers, which enables the maskless patterning of conductive carbon lines,^[^ ^2^ ^]^ has attracted considerable research interest in recent years. The resulting laser‐induced graphene (LIG), is a porous material composed of randomly stacked graphene sheets.^[^ ^3^ ^]^ LIG precursors include synthetic polymers such as polyimide, phenolic resin, and others^[^ ^4^, ^5^, ^6^, ^7^, ^8^ ^]^ as well as bio‐based precursors such as wood and other lignocellulosic materials.^[^ ^9^, ^10^ ^]^ The conversion of these precursors can be performed with different laser sources (IR, visible or UV, femtosecond, …)^[^ ^11^, ^12^, ^13^, ^14^, ^15^, ^16^, ^17^, ^18^ ^]^ and under different conditions, resulting in different morphologies and properties of the LIG produced. Laser‐induced pyrolysis is a chemical‐free process that can be easily scaled up to an industrial level using a “roll‐to‐roll” approach. The use of LIG has been demonstrated in many applications such as physical sensors (strain and pressure sensors, gas sensors, temperature, and humidity sensors),^[^ ^19^ ^]^ chemical sensors (for detecting glucose, salmonella, uric acid, tyrosine, dopamine, and others),^[^ ^20^ ^]^ actuators for soft robotics,^[^ ^21^ ^]^ and energy devices such as lithium‐ion batteries,^[^ ^22^ ^]^ supercapacitors,^[^ ^10^, ^23^ ^]^ and triboelectric nanogenerators.^[^ ^24^ ^]^

The current LIG precursors are predominantly polymer sheets, with polyimide (PI) a high performance polymer‐ being the most prevalent. In recent years, there has been a notable shift in focus from high performance synthetic polymers toward biobased precursors, particularly ligno‐cellulosic materials, such as wood or processed derivatives thereof, including paper and lignin, also in sheet form.^[^ ^9^, ^10^ ^]^ An iron‐catalyzed laser‐induced graphitization (IC‐LIG) has been proposed for engraving large‐scale electrically conductive structures on wood, based on the addition of iron‐salts‐based coatings.^[^ ^25^ ^]^ It has been shown that also non‐polymeric precursors can be laser pyrolyzed. For one metal−organic frameworks (MOFs) were carbonized with a 10.6 µm laser and used as electrodes in a supercapacitor.^[^ ^26^ ^]^ In another case a custom aromatic liquid precursor from pyrogallol and aniline was synthesized to 3D print carbon structures in a process similar to resin printing.^[^ ^27^ ^]^

Each type of precursor has its own advantages and disadvantages in terms of properties and performance for certain applications. However, one common feature of film type precursors such as PI sheets or paper is that the formed LIG is then supported and adhered to the precursor surface. Which, on its own, is not a bad property but makes it harder to create LIG on any given preexisting surface, with the aim of functionalizing it. It has been demonstrated that it is possible to create LIG by coating a surface with a photoresist consisting of a novolak an aromatic thermoplastic polymer and laser scribing it.^[^ ^28^, ^29^ ^]^ This method is even compatible with lithographic processes and allows to scribe LIG structures smaller than the laser spot diameter to be scribed.

There are several transfer techniques for LIG from the precursor surface, such as the embedding and peeling off in a cross‐linkable matrix like silicone,^[^ ^30^, ^31^, ^32^ ^]^ the transfer via adhesion with adhesives^[^ ^33^ ^]^ or hot pressing,^[^ ^34^ ^]^ among others. A recent approach for obtaining free‐standing LIG (i.e. LIG tracks not supported by any substrate) was demonstrated by using liquid nitrogen and the mismatch of the thermal expansion coefficient of LIG and the polybenzoxazine precursor.^[^ ^35^, ^36^ ^]^

This study focuses on a new class of non‐polymeric LIG precursors: dyes. More specifically, we have found that Eosin Y, a xanthene dye, is an excellent LIG precursor. Eosin Y is a synthetic dye that was first obtained 150 years ago. At the time, it was used by the famous painter Vincent Van Gogh, among others, for its brilliant color.^[^ ^37^, ^38^ ^]^ Today, this dye is used in histology for staining cells,^[^ ^39^ ^]^ photoredox catalysis^[^ ^40^ ^]^ and dye‐sensitized solar cells,^[^ ^41^ ^]^ as well as commonly used in the formulation of many commercial inks and paints. The heterocyclic rings of the xanthene and the aromatic ring attached to it make it a seemingly perfect starting point for LIG.^[^ ^3^ ^]^ A first evidence that aromatic moieties added to paper can enhance the LIG formation was presented, however the contribution from the dye and the wax in LIG formation could not be distinguished.^[^ ^42^ ^]^

Starting from an accidental discovery that some commercially available, non‐permanent markers could be carbonized with a laser, this study investigates the dye composition of those ink formulations and why certain dyes can be carbonized (and others dyes cannot). Furthermore, it explores some applications of LIG from dyes and their dispersions in various formulations.

A further aim of this work is to propose a novel methodology that we named “Paint & Scribe”, enabling to integrate LIG electrically conductive tracks, sensors, electrodes onto virtually any surface. Indeed, for dyes there is an abundance of dispersing media and formulations (inks, paints) and of dispensing/deposition techniques (with pens, brushes, markers, spray, various printers, etc.) that can be used to coat any wettable surface both at industrial/automated and craft/manual level. In brief, in “Paint & Scribe” the surface of interest can be coated with the dye formulation of choice through a suitable technique and then laser scribed.

Overall, the study marks a clear and profound difference compared to the approaches proposed so far which used polymer films and biobased materials as precursors. This also includes that the dyes and their dispersions are readily available and soluble in non‐toxic solvents like water or alcohol, unlike the polymer precursors. Furthermore, the use of dyes enables common printing and painting techniques. All of this opens many new possibilities for LIG and its application.

The first part of this contribution is about identifying the dye components of the commercially available markers through UV–vis, FTIR, and Raman spectroscopy. The identified dyes were then screened for laser‐induced pyrolysis by scribing them with both an IR and a UV laser. Although all the identified dyes have a similar structure with heterocyclic rings, quite surprisingly one dye in particular could be identified to be well pyrolyzed by the laser: Eosin Y. To further refine the hypothesis on why certain dyes could be carbonized, other xanthene dyes such as Eosin B, Rhodamine B, Rhodamine 6G and Fluorescein were laser‐scribed and screened. For these xanthene dyes the extinction coefficient at 355 nm ‐the wavelength of the UV laser used for laser scribing‐ was measured. A thermogravimetric analysis (TGA) was performed, to evidence the differences in thermal properties among the selected dyes.

The second part of the study examines the LIG from Eosin Y, investigating its sheet resistance, chemical structure and morphology in relation to the laser parameters used for scribing. A detailed analysis for one chosen laser setting is done via energy dispersive X‐ray (EDX), X‐ray photoelectron spectroscopy (XPS) and X‐ray diffraction (XRD) analysis

Furthermore, acrylic binder is used as a carrier to disperse the dye and create a carbonizable paint. The same investigations were carried out for the paint dispersion with different concentrations of Eosin Y.

In the last part some proof of concept of application of this new LIG precursor and, more in general, of the “Paint & Scribe” methodology, is demonstrated by two different approaches. The first approach shows the spray painting of a mug and laser scribing a LIG‐based temperature sensor on it. Also, the applicability of this method to integrate LIG tracks and sensors into printed flexible electronics is shown. The second approach exploits the solubility of the acrylic binder and demonstrates a novel method for creating and transferring freestanding LIG. This approach was used to fabricate a transferred temperature sensor and a stretchable conductor, made by embedding the freestanding LIG into a soft elastomer matrix. Both approaches are not possible with other polymeric precursors, as presented so far in the literature.

## 2 Results and Discussion

### 2.1 Investigation of Different Dyes and Conversion Mechanism

**Figure 1:**
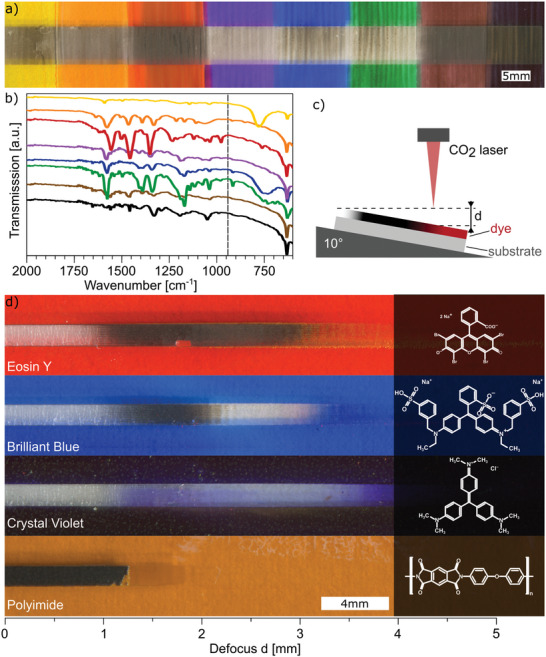
Investigation of non‐permanent pen markers and dye identification. a) Image of areas of acrylic glass colored with various Lumocolor markers irradiated with an IR laser showing carbonization and/or ablation, b) FTIR of Lumocolor markers (dashed line = IR laser sources wavenumber); c) schematic of continuous defocus d screening with a 10° wedge; d) images of areas of glass slides coated with a film of the identified dyes and polyimide (considered as a standard reference polymeric precursor of LIG) showing ablation and/or carbonization at different levels of defocus (inset: chemical structures of dyes and polyimide).

A set of non‐permanent pen markers from Staedtler (Lumocolor non‐permanent pen 315) was the starting point of the investigation. Eight different colors were applied to glass microscope slides with the markers and irradiated with a 10.6 µm IR laser rastering. As shown in **Figure** **1**a, only some of the colors are carbonized by the laser (black parts), while others were just ablated (as usual for most of the substances subjected to such a laser rastering). Red is carbonized the most, followed by yellow, orange, brown and black. Blue and green show only some traces of carbonization, while violet shows no carbonization but only ablation up to d = 4.5 mm.

The formulation of commercial inks contained in the markers can be quite complex, comprising a mixture of various dyes with several additives and other ingredients whose exact concentration is not fully disclosed. As a result, the identification of dyes present in each color and of the chemical species responsible for carbonization is not straightforward. To better study and understand the influence of each component, the commercial markers were fully characterized, and an attempt was made to identify the present dyes via spectroscopy analysis. Details on the complete spectroscopic analysis via UV–vis, ATR‐FTIR (Figure 1b), Raman spectroscopy, and the resulting identification of the dyes are reported in a dedicated section of the Supporting Information (Characterization of dyes in the commercial markers, with Figures (S0.1–S0.4) and in Figures S1 and S2, Supporting Information).

The spectroscopic characterization of the complex inks contained in the markers permitted the identification of the primary dye components, including Eosin Y, Crystal Violet and Brilliant Blue. These were subsequently employed in their pure form for further investigations. The three dyes are all used as common colorants in a variety of products. However, they are different with concerning their molecular structure (inset Figure 1d), with Eosin Y being a xanthene dye, while Crystal Violet and Brilliant Blue are triarylmethane dyes.^[^ ^43^ ^]^ Thin films of the dyes were obtained by spin coating their solutions onto glass slides. These films were scribed with the IR laser as depicted in Figure 1c, to screen their carbonization. Supporting the samples on a wedge with a 10° slope permitted the exploration of a continuous defocus^[^ ^44^ ^]^ to find the best defocusing conditions. Figure 1d displays scanned images of the pure dye films and of a polyimide sheet laser‐scribed with such a method for comparison purposes. Polyimide is considered as a standard reference polymeric precursor for LIG. Ablation occurs for all dyes up to a defocus of around *d* = 1 mm. A section with carbonization is visible for Eosin Y (≈ 1 < *d* < 3 mm) and Brilliant Blue (≈ 1 < *d* < 2 mm), whereas Crystal Violet is only ablated.

While the carbonized section of Eosin Y film was found to be electrically conductive, the carbonized Brilliant Blue did not conduct because the resulting carbon film was likely too thin for a macroscopic measurement (Figure S3, Supporting Information). Interestingly, after a section with carbonization, the Brilliant Blue dye gets ablated again at a defocus of *d* = 2 mm. Compared to Polyimide, which gets pyrolyzed from *d* = 0 (focused condition) up to around *d* = 1 mm of defocus, the carbonization of Eosin Y requires less fluence (i.e. laser energy per surface area) created by the higher defocus of the laser.

So, despite laser‐induced carbonization could also happen in the case of some triarylmethane dyes, only the xanthene dye (Eosin Y) could result in a conductive carbonized structure. The condensed aromatic structure of xanthene can be conceived as an ideal precursor for graphene and LIG, given the abundance of sp^2^ carbons and the parallels with other polymer precursors.^[^ ^3^, ^9^ ^]^

**Figure 2:**
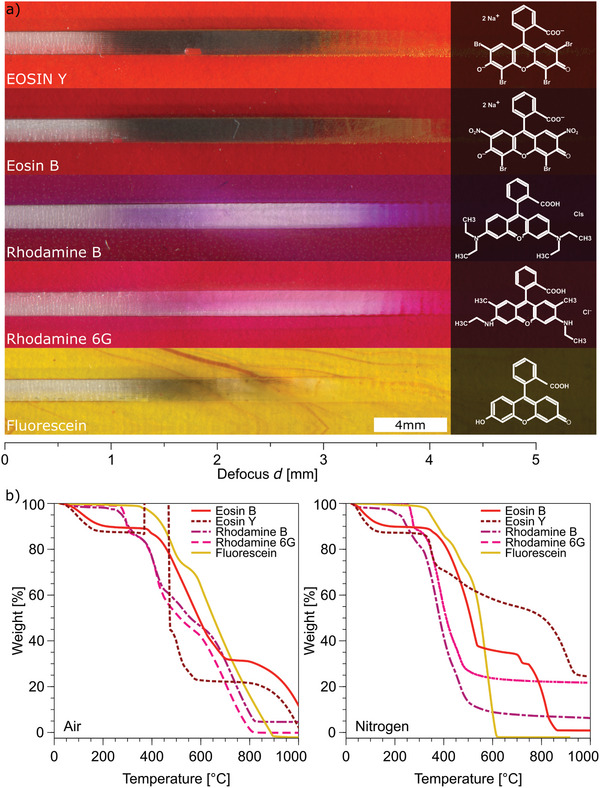
Investigation of selected xanthene dyes for laser‐induced pyrolysis. a) Images of films of selected xanthene dyes showing ablation and carbonization at different levels of defocus *d* (inset: chemical structure of xanthene dyes). b) TGA of selected dyes in air and nitrogen environment showing different weight losses.

It can be reasonably assumed that all xanthene dyes (i.e. retaining a similar xanthene unit, which is the condensed aromatic structure) could serve as effective LIG precursors. To confirm this hypothesis and to further assess the process of laser‐induced pyrolysis of dyes, the chemical structures of other xanthene dyes with comparable properties to Eosin Y were evaluated. These included: Eosin B, Rhodamine B, Rhodamine 6G, and Fluorescein (**Figure** **2**a). Eosin B demonstrated carbonization patterns similar to those observed in Eosin Y, whereas both the Rhodamines exhibited just ablation. Similar to Brilliant Blue, ablation reoccurs for the Rhodamine samples at a defocus of *d* = 2 mm. Fluorescein was ablated and transformed into a thin, non‐conductive black film at a defocus of *d* = 1 mm, exhibiting characteristics similar to those of soot.

To obtain a comprehensive representation and a more detailed analysis, all the markers and dyes (with the exception of fluorescein) were also irradiated with a UV laser (355 nm).

In comparison to IR irradiation, all marker colors aside from red exhibited lower levels of carbonization when irradiated with UV (Figure S4, Supporting Information), with respect to IR irradiation.

The pure dyes demonstrated a comparable behavior upon laser scribing with UV radiation as with IR radiation (Figure S5, Supporting Information). Eosin Y and Eosin B were carbonized, while Rhodamine 6G, Rhodamine B and Crystal Violet were ablated. Brilliant Blue was converted into a thin non‐conductive black film, resembling soot.

Although the chemical structure of these xanthene dyes is very similar, they exhibit very different behavior under laser irradiation. This invalidates the original hypothesis that polycyclic aromatic hydrocarbons, especially xanthene dyes, facilitate LIG formation. More investigations were carried out to determine the difference between these materials during laser irradiation. One possibility is that they have different optical properties and thus interact differently with the incoming laser radiation. The extinction coefficient for the dyes gives an indication of the absorption at a specific wavelength. The molar extinction coefficients at 355 nm (*ε*_355_) for the identified dyes were determined by means of UV–vis spectroscopy. Slightly lower coefficients for Crystal Violet (*ε*_355_ = 3600 м^−1^ cm^−1^) and Brilliant Blue (*ε*_355_ = 3270 м^−1^ cm^−1^) were obtained compared to Eosin Y with *ε*_355_ = 4190 м^−1^ cm^−1^.

However, UV–vis spectra of the other xanthene dyes showed that also Eosin B and Rhodamines have significant absorption bands in the spectral range of 340–360 nm (Figure S6, Supporting Information). The molar extinction coefficients *ε*_355_ were calculated for all the tested xanthene dyes (**Table** **1**). Fluorescein has the lowest extinction coefficient, followed by Eosin Y, Eosin B, and Rhodamine 6G. Among the dyes, Rhodamine B has the highest extinction coefficient (twice that of Eosin Y). Nevertheless, although Eosin B was also carbonized with both the IR and UV laser, the Rhodamines only showed ablation and no carbonization at any defocusing condition. These findings indicate that optical absorbance alone is not the key factor in laser‐induced pyrolysis of dyes. This assertion is likely applicable to all LIG precursors, though further investigation is necessary to confirm it.

**Table 1:** Molar extinction coefficients ε_355_ for the tested xanthene dyes, as obtained from UV–vis Spectra.

Dye [10^−4^ M water solution]	*ε*_355_ [м^−1^ cm^−1^]	R^2^		Carbonization
Eosin B	6480		0.99	Yes
Eosin Y	4190		0.99	Yes
Fluorescein	400		1	No
Rhodamine B	10790		0.99	No
Rhodamine 6G	5500		0.99	No

Given the high fluence values of the laser irradiation and the high temperature (in the order of 3000K)^[^ ^45^ ^]^ at the focus spot, it is evident that other properties such as the thermal stability of the precursor should play an important role. This phenomenon has also been observed in bioderived LIG precursors but was attributed to the chemical structure, as in the case of lignin and cellulose.^[^ ^9^, ^10^ ^]^

To study thermal stability, thermogravimetric analysis (TGA) was conducted on the xanthene dyes in different atmospheres (air and nitrogen). They show a distinct difference above 600°C (Figure 2b). This variation in weight loss could potentially be the critical factor for determining LIG formation. In the range 0–300°C, the investigated xanthene dyes share a similar behavior in inert atmosphere (nitrogen) at a heating rate of 10°C min^−1^ and in air at a heating rate of 100°C min^−1^. After initial small weight loss due to dehydration, Fluorescein starts to decompose at 330°C, while Rhodamines at 220 – 250°C. Eosins show instead a more significant initial mass loss, possibly ascribable to dehydration together with partial debromination.^[^ ^46^ ^]^ Above 300°C, Fluorescein rapidly reaches complete decomposition under nitrogen flow (within 600°C), while decomposes slower in air (within 900°C). The Rhodamines decompose above 800°C, leaving ≈ 10% mass residue. When under nitrogen flow a residue of 25% for Rhodamine B and 5–10% for Rhodamine 6G is left. Eosin Y and Eosin B have different thermal behavior, but both show significant mass loss between 300 and 600°C. These two dyes share higher stability compared to other xanthene dyes above 600°C, retaining a residual weight above 20% and undergoing significant or complete decomposition only after 900°C (nitrogen) and 1000°C (air), respectively.

Overall, the measurements in air at a higher heating rate showed a shift to higher decomposition temperatures with respect to those in nitrogen at a lower heating rate, without significant changes in the decomposition path for most of the dyes. This effect can be ascribed to a slower equilibration of the sample with the external furnace temperature, when higher temperature gradients are applied.^[^ ^47^ ^]^ Moreover, the activation energy is reported to directly vary with heating rate.^[^ ^48^ ^]^ Furthermore, under nitrogen flow, smaller mass loss and some final residue are found with respect to air, for Rhodamines and Eosin B. TGA of Eosin B in air at a heating rate of 10°C min^−1^ (not reported) showed an intermediate thermal behavior, i.e. total decomposition occurring within 900°C, confirming that residual mass is found only in inert atmosphere, despite of the heating rate.

The actual conditions present during laser‐induced pyrolysis are difficult to replicate in a controlled TGA experiment. However, they are situated somewhere between inert and air surroundings since the process also takes place within the material, in the absence of an external atmosphere^[^ ^45^, ^49^, ^50^ ^]^ and a glowing plasma is observed during laser scribing. The steps of the decomposition pathway are dependent upon the binding energies of the various functional groups that are attached to the xanthene core. Additionally, the degree of functionalization and the molecular weight are contributing factors. The latter is highest for Eosins, which, in fact, have the highest thermal stability in both operating conditions.

The improved thermal stability of Eosins seems to be the key factor in laser‐induced pyrolysis allowing for enough material to be converted in LIG. In the case of low thermal stability, the material is already completely thermally decomposed before the onset of laser‐induced pyrolysis and thus only ablation is observed.

### 2.2 LIG from EOSIN Y

Eosin Y and Eosin B were identified as the most suitable LIG precursors from the set of investigated dyes. Eosin Y was chosen for further studies in which an IR laser was used for laser scribing. The dye was dissolved in methanol and labeled with increasing Eosin Y concentration (M2, M4, and M6, as defined in the Experimental Section).

The dye solution was spin‐coated, resulting in films of pure dye with varying thickness *t* depending on the initial concentration (*t*_M2_ = (630 ± 140) nm, *t*_M4_ = (1700 ± 100) nm, *t*_M6_ = (2500 ± 200) nm). The dye concentration solely affected the film thickness as the solvent evaporated entirely.

**Figure 3:**
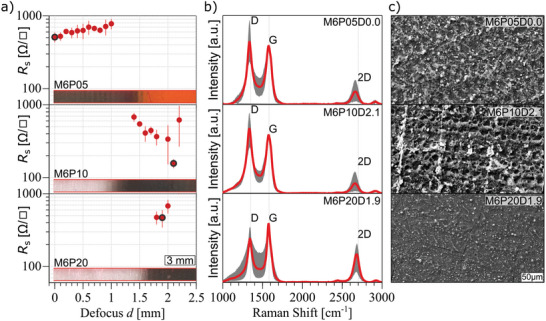
Characterization of LIG from Eosin Y films obtained from dye solutions at maximum concentration M6 and scribed with IR laser at *P* = 5% (top), 10% (middle), and 20% (bottom). a) Sheet resistance as a function of defocus with insets showing the corresponding optical images of the scribed samples. In b) average Raman spectra with standard deviations (shaded areas) and c) SEM images of the samples showing the lowest sheet resistance, marked in black in a).

To determine the optimal IR laser parameters for carbonization, a screening of the fluence space via continuous defocus^[^ ^44^ ^]^ was done. In addition, the LIG sheet resistance was measured. The thickness of the dye films played a crucial role in the resulting sheet resistance. Films M2 and M4 both exhibited sheet resistances around *R*_s_ = 1000 Ω/□, which can be related to low dye layer thickness. The sheet resistance values for certain defocus steps and powers for the M6 sample were measured and are plotted in **Figure** **3**a up to a maximum of *R*_s_ = 1000 Ω/□.

The optical result of the continuous defocus screening for M6 can be seen in the inset of Figure 3a. For low power (*P* = 5%) the carbonization starts at the focused condition and stops at around *d* = 1.5 mm. This marks the threshold that matches the necessary fluence for laser‐induced pyrolysis. Lower fluence (higher defocus) does not produce any effect. However, the sample turns already “non‐conductive” (*R*_s_ > 1000 Ω/□) at *d* = 1 mm. For higher power the inset shows that in a focus condition the material is completely ablated. This is caused by excessive laser fluence for this layer thickness: not enough material is present to sustain the ablation and carbonization. As the defocus increases and the laser fluence decreases, carbonization sets in at around *d* = 1 mm for *P* = 10% and *d* = 1.5 mm for *P* = 20%, and the samples turn conductive. For *P* = 10% a minimum sheet resistance of *R*_s_ = 156 ± 7 Ω/□ is measured at a defocus of *d* = 2.1 mm. In the case of *P* = 20% the conductive area is significantly smaller resulting from the overall higher fluence and therefore higher ablation.

The thickness of the LIG layer for M6P10D2.1 (*t* = 3590 ± 1400 nm) was measured on SEM images of cross sections prepared with a Focused Ion Beam (FIB) (Figure S7, Supporting Information). The increase in thickness of the LIG layer compared to the dye layer before laser‐induced pyrolysis can be explained by the porosity of the LIG layer induced by the degassing during laser scribing.^[^ ^3^, ^45^ ^]^ Using the thickness value, sheet resistance and Equation (1) the conductivity is estimated to be 21 ± 9 S cm^−1^, a value comparable with the conductivity of polyimide‐derived LIG.^[^ ^3^ ^]^

The LIG resulting from Eosin Y (M6) is only a few micrometers thick, which results in a relatively high sheet resistance compared to other examples from the literature.^[^ ^51^, ^52^ ^]^ In these cases, the LIG thickness is several hundred micrometers, which makes direct comparison difficult. The LIG thickness can be increased by mixing the dye with an acrylic binder, which is discussed in the next section.

The minimum sheet resistance for the different films and different power settings are listed in **Table** **2** together with the corresponding defocus setting.

**Table 2:** Lowest sheet resistance values of LIG obtained from Eosin Y films with varying concentrations (M2, M4, M6), and laser scribing powers of *P* = 5, 10, 20% and defocus *d*.

	*P* = 5%	*P* = 10%	*P* = 20%
**M2**	2400 ± 100 Ω/□ @ *d* = 0.4 mm	2200 ± 200 Ω/□ @ *d* = 1.5 mm	n.c.
**M4**	860 ± 40 Ω/□ @ *d* = 0.1 mm	620 ± 20 Ω/□ @ *d* = 1.6 mm	900 ± 140 Ω/□ @ *d* = 1.8 mm
**M6**	510 ± 25 Ω/□ @ *d* = 0.0 mm	156 ± 7 Ω/□ @ *d* = 2.1 mm	470 ± 40 Ω/□ @ *d* = 1.9 mm

Raman spectra collected from the surface of the carbonized samples show the presence of the typical graphene signals (Figure 3b): G band (primary graphene mode), representing the vibrations of planar sp^2^ bonded carbons; D band (disorder or defect band), representing the ring breathing mode from sp^2^ carbon rings, if adjacent to a graphene edges or defects; and 2D band, which is the second order of the D‐band, but does not need defects to be activated.^[^ ^53^ ^]^ The latter band is used to determine graphene layer thickness. The average Raman spectra of samples with the lowest sheet resistance for each power setting are plotted in Figure 3b. The I_D_/I_G_ ratio decreases with power from 1.0 ± 0.4 for *P* = 5% to 0.7 ± 0.1 for *P* = 20%. The I_2D_/I_G_ ratio is 0.2 ± 0.2 for lower power (*P* = 5% and 10%) and increases for *P* = 20% to 0.5 ± 0.2. Although a lower number of defects is evidenced for samples obtained at P = 20% with respect to those at lower power, their sheet resistance is higher. The peak position and the full width at half maximum (FWHM) can be found in the Supporting Information (Table S1, Supporting Information). The Raman spectra for the carbonized M2 and M4 can be found in Supporting Information (Figure S8, Supporting Information) and show I_D_/I_G_ ≈ 1 and I_2D_/I_G_ ≈ 0.5.

Comparing the spectra of one of the less conductive samples (M2P10D1.5) with those of one of the more conductive samples (M6P10D2.1), we can see that in the first case the spectrum shows a peak at 1100 cm^−1^ due to CH bending, which is absent in the second case.^[^ ^54^ ^]^ This band (1100–1200 cm^−1^) is also weakly present in the original Eosin Y (Figure S9, Supporting Information). This indicates the presence of sp^3^‐hybridized carbons, and thus non‐continuous and homogeneous LIG layers. However minor bands located at ≈ 1100–1200 have been reported in the literature as also associated with phonon‐defect processes in graphene″.^[^ ^55^ ^]^

The single‐point Raman spectra only give very limited information about the sample as the sampling area is very localized. By collecting 2D Raman maps over a larger area a more complete picture can be retrieved. Raman 2D maps of M2P10D1.5 and M6P10D2.1 samples are shown in Figure S10 (Supporting Information). The ranges of the I_D_/I_G_ and I_2D_/I_G_ values obtained through the whole mapped areas are reported in **Table** **3** for each LIG sample. Typical graphene bands are found in all the investigated samples, together with other signals detected in low conductive samples, such as M2P10D15, and ascribable to Eosin Y as discussed before. However, the overall ratios of I_D_/I_G_ and particularly of I_2D_/I_G_ are smaller than in the case of single‐point spectra, which means that the graphenic areas are rather localized.

**Table 3:** Sheet resistance values and Raman band ratios I_D_/I_G_ and I_2D_/I_G_ for LIG obtained from Eosin Y thin films at different thickness/dye concentration (MX), laser setting: power (PX [%]), defocusing (DX [mm]), sorted from lowest to highest sheet resistance.

Sample	Laser Setting	Sheet Resistance [Ω/□]	I_D_/I_G_	I_2D_/I_G_
**M6**	P10D2.1	157 ± 7	0.4‐0.6	0.0‐0.1
	P20D1.9	469 ± 41	0.5‐0.8	0.1‐0.9
	P5D0	510 ± 25	0.5‐0.7	0.0‐0.1
**M4**	P10D16	623 ± 17	0.5‐1	0.0‐0.5
	P5D01	860 ± 38	0.5‐0.7	0.0‐0.1
**M2**	P10D1.5	2172 ± 220	0.6‐1	0.0‐0.3
	P5D04	2412 ± 107	0.6‐0.9	0.0‐0.2

The I_D_/I_G_ ratio follows a directly proportional trend with respect to the LIG's sheet resistance, that is, with increasing sheet resistance, the I_D_/I_G_ values are increasing. This confirms that more homogeneous and conductive LIG is produced when less disorder/defects are present. Low I_2D_/I_G_ are usually found in the case of multiple graphene layers as in graphite (high thickness). Here, low I_2D_/I_G_ values are summarily found in all the investigated LIG samples, indicating that stacked graphene layers are present, even if no continuous and homogenous structure forms in all scribing conditions, apparently. It is to be noted that the band's signal to noise ratio (especially of 2D band) is lower when extracted from maps instead of single measurements, due to different operating conditions (laser power, accumulations and exposure time).

SEM imaging of laser scribed Eosin Y films revealed that different LIG morphologies are obtained depending on the thickness and laser scribing parameters (Figure 3c, SEM of selected samples with low sheet resistance). In the case of thick M6 films the dye layer is turned into a mostly continuous carbon structure when scribed with low power (*P* = 5%). Increasing the power to *P* = 10% reveals an uninterrupted porous structure, with some superficial brighter dots, and irregular micrometric pores. This is in agreement with the particularly low sheet resistance that was measured for this setting (the lowest in the series).

**Figure 4:**
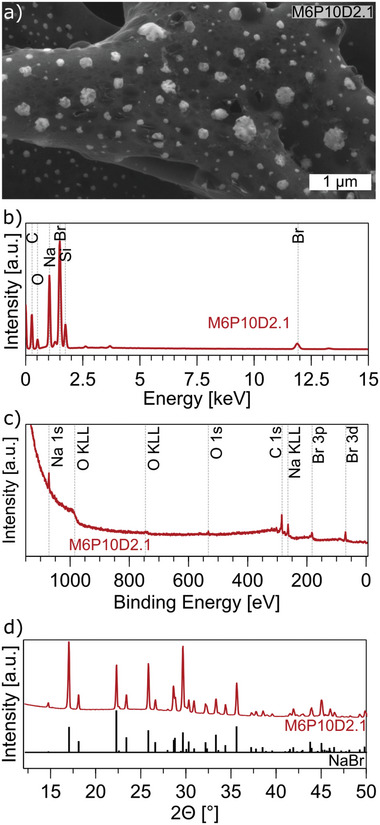
Detailed characterization of LIG from M6P10D2.1. a) High magnification SEM images showing bright spots on the porous carbon structure, b) EDX, c) XPS, d) XRD showing characteristic peaks of NaBr crystals (PDF#15‐0010).^[^ ^60^ ^]^

High‐magnification SEM images show a decoration of the LIG with small bright particles (**Figure** **4**a). It is reasonable to assume that the particles could be NaBr crystals formed as byproducts of Eosin Y degradation during laser‐induced pyrolysis. It has already been shown that decoration of LIG with metallic nanoparticles can be achieved by incorporating metal salts into the precursor material.^[^ ^56^, ^57^, ^58^, ^59^ ^]^ Conversely, when a high laser power (*P* = 20%) is employed, the dye film undergoes additional ablation, resulting in the formation of unconnected matter and, in turn, in a larger sheet resistance. The thin M2 samples are turned into disconnected aggregates of micrometric objects (Figure S11, Supporting Information), while the intermediate thickness M4 samples show both disconnected micrometric objects and a dotted semi‐interconnected structure (Figure S12, Supporting Information). In both images, the surface of the glass substrate exhibits cracking due to the laser irradiation.

To gain a more precise understanding of the elemental composition, energy dispersive X‐ray analysis (EDX) was conducted on different areas of the thick M6 samples. For the low‐resistance M6P10D2.1 sample C, O, Na, Br, and traces of Si, Al, Mg, and K are detected (Figure 4b). The Br and Na are presumed to originate from the bright particles, likely NaBr crystals forming from the Eosin Y. The EDX analysis of the bright white spots observed in the SEM images revealed a 1:1 ratio of Na and Br, thereby validating the assumption that NaBr crystals are decorating the LIG (Figure S13, Supporting Information). As regards Si and other trace elements they likely come from the glass substrate as the penetration depth of EDX is in the range of micrometers. The atomic percentage of these trace elements exhibited an increasing trend with a reduction in Eosin Y film thickness, reaching its highest value in the thin M2 film (Figure S14; Table S2, Supporting Information). This observation is consistent with the hypothesis that the lower amount of carbonized material and lower film thickness for these films likely contributed to this result.

The chemical composition of the carbonized M6 films was further investigated by X‐ray photoelectron spectroscopy (XPS). The spectra show peaks for Na, O, C, and Br (Figure 4b; Figure S15, Supporting Information). Samples obtained with a power of *P* = 5% and 10% show a similar content. The carbon content is the lowest (≈50 at%) for *P* = 20% which makes sense given the high sheet resistance and discontinuous carbon film observed in the SEM images. The Na content is the lowest for the sample with low sheet resistance (M6P10D2.1) while the Br content increases from 6.35 at% to 8.76 at% with increasing power. The oxygen content is ≈ 6 at% except for the high power (*P* = 20%) sample with 26.58 at%.

Figure 4d displays the X‐ray diffraction (XRD) spectrum of M6P10D2.1 that depicts distinct peaks attributed to NaBr crystals. However, it is noteworthy that there is a conspicuous absence of any significant peak for LIG. This can be attributed to its low crystallinity, which is also evident in the Raman spectroscopy data (I_D_/I_G_ >1) and SEM images.

### 2.3 LIG from Acrylic Paint

Dyes are commonly used as dispersions in other media, such as coatings and paints. The dispersion of Eosins (which were identified as good LIG precursors) permits the carbonization of these paints, which would otherwise be considered non‐carbonizable materials. Furthermore, we envision the use of Eosin‐based inks and paints in a “Paint & Scribe” approach. Target surfaces are painted with a suitable ink/paint and then laser scribed, enabling the integration of electrically conductive LIG tracks, sensors, electrodes onto virtually any paintable surface. The subsequent section introduces the methodology of the “Paint & Scribe” approach and presents some proofs of concept. This section, in further detail, investigates the dispersion of Eosin Y in a representative medium, namely acrylic binder.

The investigation on Eosin Y /acrylic binder was the same as for the pure dye films and started with screening of the laser fluence space via continuous defocus. Figure S16 (Supporting Information) shows that the pure dried acrylic binder is just ablated during scribing. Thus, the acrylic binder is not per se a LIG precursors, as it could not be carbonized via laser scribing. By mixing the acrylic binder with Eosin Y, laser‐induced pyrolization can be enabled (Figure S16, Supporting Information). To investigate the influence of the dye on the pyrolysis process, different concentrations were mixed with the acrylic binder. The concentration of the dye in the initial solution affects the dye concentration in the resulting film, once it is dried. The films were labeled AH1, AH2, and AH3 with increasing Eosin Y concentration (see Experimental Section for details).

**Figure 5:**
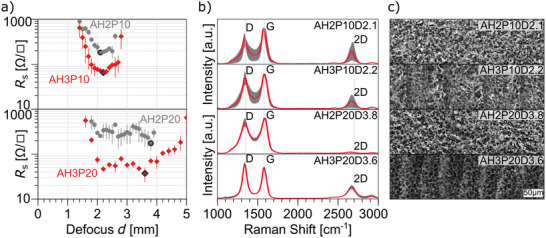
Characterization of LIG from Eosin Y/Acrylic binder films obtained from dye dispersion at two different concentrations AH2, AH3, upon IR laser scribing at *P* = 10, 20%. a) sheet resistance as a function of defocus. Marked samples in black, representing the lowest sheet resistance per each series, are further investigated with b) average Raman spectra, c) SEM images.

The film thicknesses for the different dye concentrations are *t*_AH1_ = 4900 ± 700 nm, *t*_AH2_ = 6800 ± 750 nm, *t*_AH3_ = 11200 ± 1700 nm. Again, three representative laser power values were investigated for laser scribing, *P* = 5, 10, 20%. The result for the sheet resistance investigation of the fluence space is shown in **Figure** **5**a. The low concentration of Eosin Y in AH1 films and all concentrations scribed with a low power (*P* = 5%) yielded LIG with a sheet resistance *R*_s_ > 1000 Ω/□ for all settings and were not plotted. It can be observed that the minimum sheet resistance decreases with an increase in power and concentration. The sheet resistance of AH2 is higher than that of AH3, due to the lower dye concentration in the former. While for AH3P10D2.1 a sheet resistance of *R*_s_ = 180 ± 10 Ω/□ is measured, for AH3P20D3.6 the sheet resistance decreases to *R*_s_ = 37 ± 13 Ω/□. This indicates that an increase in power has a significant impact on the sheet resistance, with a factor of ≈5. This is due to the thickness of the precursor film, as a thinner film would be ablated, similar to the case of the pure dye.

The thickness of the LIG layer for AH3P20D3.6 (*t* = 11280 ± 3570 nm) was measured on SEM images of FIB‐prepared cross sections, as described before (Figure S17, Supporting Information). In contrast to the carbonized pure dye layer, the thickness of the suspension of acrylic and dye stayed similar before and after laser scribing. This is probably due to the lower amount of carbonizable material (dye) in the acrylic film. Using the thickness value, sheet resistance and equation 1 the conductivity was estimated to be 34 ± 20 S cm^−1^. This value is comparable with the result from the pure dye layer and of polyimide‐derived LIG.^[^ ^3^ ^]^

The peak position and the full width at half maximum (FWHM) can be found in the Supporting Information (Table S4, Supporting Information). Average Raman spectra of samples showing the lowest sheet resistance for AH2 and AH3 (marked in black in plots of Figure 5a) are plotted in Figure 5b. Typical bands associated with LIG are present and an I_D_/I_G_ ratio of 0.7 ± 0.2 and 0.8 ± 0.2 were measured for AH2 and AH3 at *P* = 10%, respectively. For *P* = 20% the ratios do not change drastically: 0.8 ± 0.1 and 1.1 ± 0.1 for AH2 and AH3, respectively. I_2D_/I_G_ ratios are 0.4 ± 0.2 and 0.1 ± 0.2 for AH2 and AH3 at *P* = 10%, respectively. These values are comparable with the ones from the pure dye films (I_D_/I_G_ ≈ 1) showing the lowest sheet resistance. A different behavior can be observed for the I_2D_/I_G_ ratios, as in the case of AH2P20D3.8 where the 2D band nearly disappears. No good explanation for this could be found, except for local anomalies.

Since the local measurements vary a lot with the measurement position, a better statement can be made with 2D maps of the Raman spectra collected over a larger area, as done in the pure dyes case. Raman 2D maps of AH1P20D2.0 and AH3P20D3.65 samples are shown in Figure S18 (Supporting Information). The ranges of the I_D_/I_G_ and I_2D_/I_G_ values obtained through the whole mapped areas are reported in **Table** **4** for each LIG sample.

**Table 4:** Sheet resistance values and Raman band ratios I_D_/I_G_ and I_2D_/I_G_ for LIG obtained from Eosin Y/Acrylic binder films at different dye concentration (AHX), laser settings: power (PX [%]), defocusing (DX [mm]), sorted from lowest to highest sheet resistance.

Sample	Laser Setting	Sheet Resistance [Ω/□]	I_D_/I_G_	I_2D_/I_G_
**AH3**	P20D3.6	37 ± 14	0.5‐0.6	0.0‐0.2
	P10D2.2	65 ± 6	0.5‐0.6	0.0‐0.2
**AH2**	P20D3.8	174 ± 55	0.5‐0.6	0.0‐0.1
	P10D2.1	183 ± 13	0.5‐0.6	0.0‐0.1
**AH1**	P20D20	979 ± 163	0.6‐0.9	0.1‐0.4
	P10D21	4872 ± 923	0.5‐0.7	0.0‐0.1

Typical graphene bands are found in all the investigated samples. The I_D_/I_G_ ratio ranges from 0.5 to 0.6 for more conductive samples, and slightly increases for less conductive LIGs. The lower variation in the I_D_/I_G_ ratio in the AH series compared to the M series indicates that the LIG produced from the acrylic mixture (AH) is more homogeneous. As in the case of pure dyes, the I_D_/I_G_ and especially the I_2D_/I_G_ ratios in the maps are lower than in the single‐point spectra. It is to be noted that the band's signal to noise ratio (especially of 2D band) is lower when extracted from maps instead of single measurements, due to different operating conditions (laser power, accumulations, and exposure time).

SEM images of the selected samples are shown in Figure 5c showing a multi‐layered porous structure of LIG. With a defocus ranging from *d* = 2 to *d* = 4 mm, higher laser power and thicker films produced a more continuous and regular 3D structure. In the same defocus conditions (around *d* = 2 mm), AH3P20D3.6 sample gave better graphitization with respect to M6P20D1.9. This indicates that a laser power of 20% is too high for samples with sole Eosin Y, probably because of the low thickness as already discussed. However, it produces LIG with better performances in thicker AH paints. It was hence hypothesized that acrylic binder acts as a ‘glue’ that keeps the precursor molecules closely packed. Furthermore, evidence was found that the dye also acts as a sort of catalyst or seeding point for further carbonization of the acrylic binder itself, resulting in much denser films as compared to films with dye only (M6P10D2.1, Figure 3c). Laser‐induced pyrolysis of the AH1 samples with the lowest concentration of Eosin Y results in disconnected micrometric objects (Figure S19, Supporting Information), while AH2 samples show a superficial network similar to AH3. The morphologies observed in SEM reflect the observed trend in sheet resistance where the highest concentration of dye results in the best conductive film.

**Figure 6:**
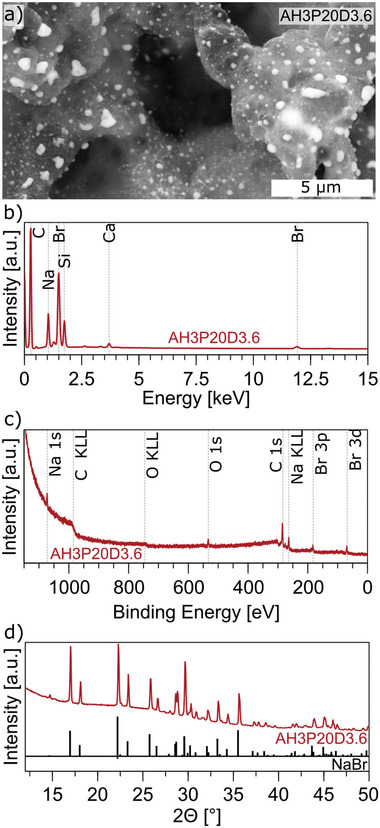
Detailed characterization of LIG from AH3P20D3.6. a) High magnification SEM images showing bright spots on the porous carbon structure, b) EDX, c) XPS, d) XRD of showing characteristic peaks of NaBr crystals (PDF#15‐0010).^[^ ^60^ ^]^

Similar to the carbonized pure dye films, high magnification SEM images show an even more prominent decoration with bright white spots (**Figure** **6**a).

EDX was carried out on different areas of AH3P20D3.6 and AH1P10D2.1. After C and O, other trace elements typical of glass substrates were detected. Br and Na were detected in AH3 samples (Figure 6b), even in a wide spectrum area, but little Br was found in AH1 samples (Figure S20, Supporting Information).

Investigations with XPS show similar results as obtained before with the pure dye films. The carbon content is the lowest for AH1P20D2.0 (64 at%) and increases to 81 at% for AH3P20D3.6 (Figure 6c). The surface oxygen content decreases with increasing dye concentration from ≈ 16 at% down to 9 at% (Figure S21; Table S6, Supporting Information).

Figure 6d displays the XRD spectrum of AH3P20D3.6, which once again exhibits the characteristic peaks corresponding to NaBr crystals. Notably, no peaks indicative of carbon were observed.

Another approach to the characterization of carbon materials in bulk involves the use of thermogravimetric analysis (TGA), a method that has been demonstrated to provide both qualitative and quantitative analysis of different carbon species, including rGO, GO, graphene, and graphite. The TGA allows for the identification of these species by measuring the temperature of maximum mass decomposition rates T_max_, and the positions of the peaks in the resulting thermogravimetric curve. Graphite, being the most stable form, has the highest T_max_, while graphene, due to the absence of van der Waals forces, has a lower T_max_. The introduction of additional defects into graphene further reduces its T_max_, as observed in the case of GO. This phenomenon can be attributed to the presence of non‐graphitic sp^3^ hybridized carbon and functional oxygen groups (CO, OH).^[^ ^61^ ^]^

TGA analysis of the LIG from acrylic binder and Eosin Y (AH3) allows to gather more information on the nature of the produced LIG (Figure S22, Supporting Information). Derivative Thermogravimetry (DTG) shows three main decompositions with two smaller ones at 358 and 490 °C possibly ascribable to CO and OH functionalities, respectively.^[^ ^62^ ^]^

A larger peak is observed at the maximum temperature of T_max_ = 614 °C related to the sp^2^ and sp^3^ defects.^[^ ^61^ ^]^ This indicates that the LIG from acrylic binder and Eosin Y (AH3) is most likely rGO with a low number of defects as T_max_ is above 600 °C.

As a comparison, the TGA of LIG from polyimide^[^ ^33^ ^]^ (Figure S23, Supporting Information) shows a curve similar to that of graphene slightly shifted to lower temperatures with T_max_ = 689 °C, indicating a small amount of non‐graphitic sp^3^ hybridized carbon.

To further illustrate the carbonization process of acrylic paint, a different acrylic binder (Aqua‐Cryl CFB) was combined with Eosin Y. Laser scribing of Eosin Y/ acrylic binder led again to the creation of LIG, a phenomenon that is not observable when the acrylic binder alone is scribed (Figure S24, Supporting Information). This evidence strongly suggests that the formation of LIG in the dispersing medium is solely dependent on the utilized dye, which serves as the LIG precursor.

### 2.4 ”Paint & Scribe”: Proofs of Concept

**Figure 7:**
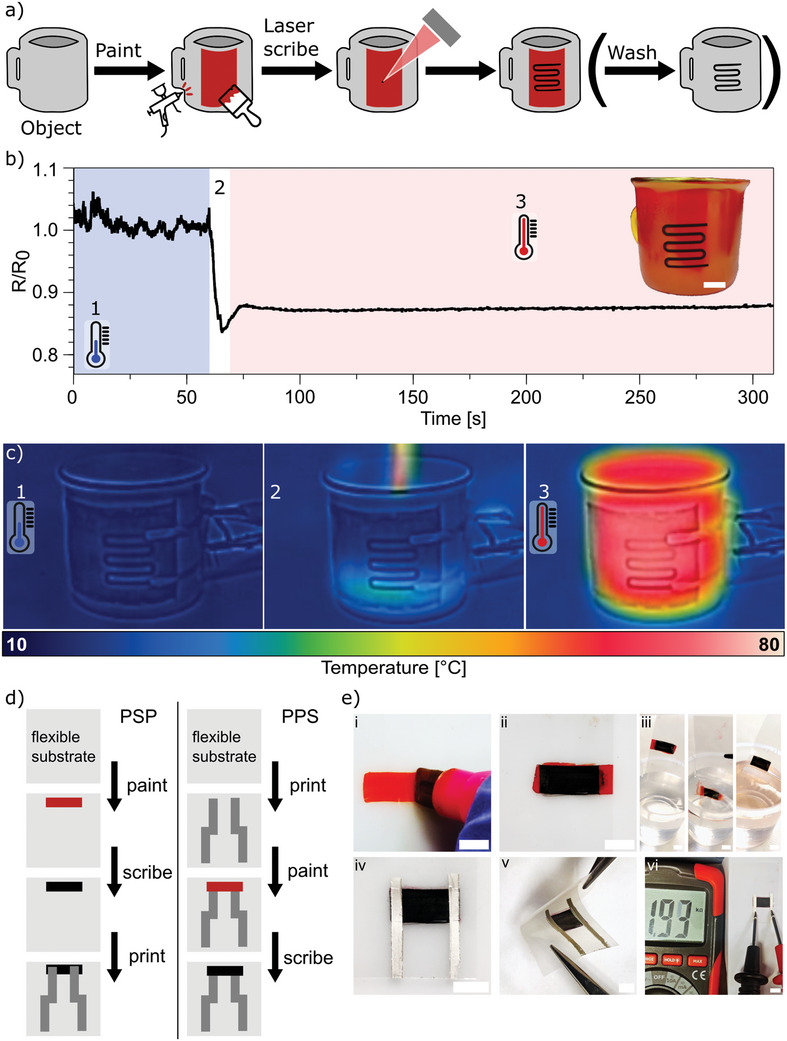
a) Schematic illustration of the “Paint & Scribe” methodology on a generic object (mug) with an optional step of removing the paint by washing. b) Change of resistance (R/R0) of the LIG thermistor when hot water is poured into the mug; inset: image of the painted and scribed mug (scale bar = 10 mm) c) Thermal camera images of the mug filled with cold water (1), being filled with hot water (2) and the heated mug (3) d) Schematic illustration of the methodology for integration of “Paint & Scribe” with printed and flexible electronics showing the two approaches, “Paint, Scribe and Print” (PSP) and “Print, Paint, and Scribe” (PPS). e) Images of the PSP variant to integrate LIG with printed silver tracks on a PET foil substrate (scale bar = 5 mm), showing the painting with a marker (i), laser scribing (ii), washing away the ink in water (iii), printing of silver tracks (iv), flexing of the sample (v) and electrical connection (vi).

The use of dye solution or dispersion in a suitable medium enables the coating of any wettable surface with a layer that serves as a carbonization precursor for laser‐induced pyrolysis, that is used to scribe conductive LIG tracks. We called this approach *“Paint & Scribe”*: the general idea and steps are schematized in **Figure** **7**a, taking as an example of target object/substrate a mug. The object is first painted with a suitable formulation of Eosin dye in a dispersing medium. The painting can be carried out with any manual or automated technique: a pen, a marker, a brush, an airbrush, screen or ink‐jet printing, or even spin or dip coating. This approach can be customized by tuning the exact formulation of the ink/paint for the selected technique and to ensure proper wettability and adhesion to the target substrate. Then, laser scribing with IR, UV or visible laser source enables the laser‐induced pyrolysis according to a predetermined pattern. The remaining paint in non‐scribed parts can be either left on the substrate or removed by washing; typically, water is used in the case of the non‐permanent marker ink, but the actual solvent can vary depending on the ink formulation.

This general approach was demonstrated by spray coating a mug with the commercially available Lumocolor^®^ ink from the markers. A LIG‐based resistive temperature sensor is then laser scribed with an UV laser (Figure 7a). The initial resistance at (1) (*R*_0_ = 23.5 ± 0.7 kΩ, mug filled with cold water) begins to decrease when hot water is poured into the mug (2) (Figure 7b). The resistance of the thermistor decreases to *R*/*R*_0_ = 0.82 for the heated mug (3) and exhibited the typical negative temperature coefficient (NTC) upon heating.^[^ ^63^ ^]^ The thermal camera images in Figure 7c shows the corresponding temperature change on the surface of the mug.

In addition to representing an appropriate method for scribing LIG tracks on diverse objects and bulk materials, the proposed “Paint & Scribe” approach may also be a viable technique for integration of LIG with printed and flexible electronics. It is worth noting that the typical substrates employed in printed and flexible electronics, including glass, PCB, paper, polyethylene terephthalate (PET), polyethylene naphthalate (PEN), polydimethylsiloxane (PDMS) and other silicone rubbers, thermoplastic polyurethane (TPU)^[^ ^64^, ^65^ ^]^ are typically not suitable for carbonization via laser‐induced pyrolysis. Figure 7d illustrates two potential methodologies for integrating LIG tracks with printed and flexible electronics. The first variant begins with the painting of the substrate with the ink or paint, followed by laser‐induced pyrolysis. Subsequently, the paint may be removed (if desired) or allowed to remain on the substrate. Additional components may be printed on the substrate and electrically connected to the LIG. This variant was designated “Paint, Scribe and Print” (PSP). In contrast, the alternative approach involves printing the substrate first and then applying the carbonizable paint on top of the printed structures. This variant is designated as “Print, Paint, and Scribe” (PPS). Both variants have advantages and disadvantages. The PSP variant ensures a good electrical connection by partly printing over the LIG, but the application of certain printing techniques can be limited. For example, screen printing could damage the LIG. The PPS variant, on the other hand, allows for all printing techniques to be applied initially, but this may result in issues with the electrical connection, as not all of the paint may be fully carbonized. This could potentially create a non‐conducting barrier between the printed layer and the LIG. Moreover, the use of a solvent for the removal of paint could be restricted in certain cases. In Figure 7e a demonstration of the PSP variant for the integration with printed and flexible electronics is shown. The PPS variant is shown in Figure S25 (Supporting Information).

**Figure 8:**
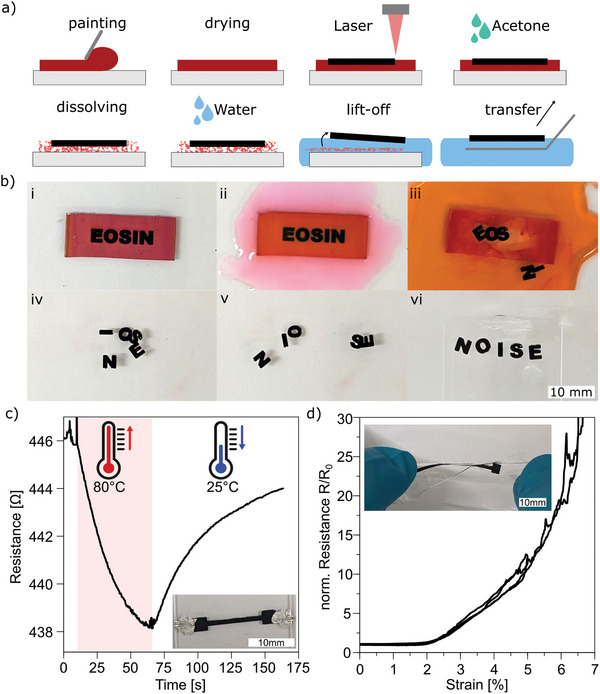
a) Schematic showing the creation and transfer of free‐standing LIG from an Eosin Y/acrylic binder precursor (detailed description in text). b) Pictures of LIG alphabet soup demonstrating the lift‐off transfer of free‐standing LIG from acrylic paint creating the anagram of EOSIN “NOISE”. c) Thermistor from free‐standing LIG transferred onto a glass slide heated to 80°C and cooled down to room temperature. d) Stretchable conductor made from free‐standing LIG transferred and embedded in PDMS.

The use of a dispersion of Eosin Y and acrylic binder allows for the creation of a soluble LIG paint precursor, different from polymer precursors which are hardly soluble in many solvents. This soluble paint facilitates the obtainment and transfer of freestanding LIG (**Figure** **8**a). Following the painting and drying of the dispersion, laser pyrolysis is performed. The non‐carbonized areas of the paint can be dissolved by soaking it with acetone and subsequent washing and submerging in water. This causes the LIG to lift off from the substrate and float on water surface. The free‐standing LIG can then be collected from the water bath and be transferred onto target surfaces. This is illustrated in Figure 8b, which depicts laser‐scribing the word “EOSIN” onto an acrylic paint‐coated glass substrate (i) and dissolving the acrylic paint with acetone (ii). Following the washing with deionized water (iii), the floating letters creating an alphabet soup are rearranged (iv, v) and recollected onto a glass substrate to spell the anagram “NOISE” (vi).

The same process was employed to transfer a LIG track onto a glass substrate, which was utilized as a temperature sensor to demonstrate the typical NTC behavior of LIG (Figure 8c).^[^ ^63^ ^]^ Additionally, the transfer process facilitates embedding of the LIG in a stretchable matrix (PDMS) to create stretchable conductors (Figure 8d). In contrast to the state of the art of embedding LIG, where the LIG created on a precursor substrate is coated by a liquid mixture, which is then crosslinked and peeled off,^[^ ^31^ ^]^ this new approach utilizes the freestanding LIG for a transfer. This allows the LIG to be transferred or adhered to almost any target surface.

In comparison to a recent approach for freestanding LIG, which was demonstrated by using liquid nitrogen and the mismatch of the thermal expansion coefficient of LIG and the polybenzoxazine precursor,^[^ ^35^, ^36^ ^]^ our approach features a low‐cost, user‐friendly, and adaptable precursor. Additionally, the acetone‐ and water‐based lift‐off process enables a more precise and regulated process.

## 3 Conclusion

The discovery of laser‐pyrolyzable dyes has led to the development of a novel approach to the creation of LIG. Commercially available markers containing specific dyes can be carbonized via laser‐induced pyrolysis, resulting in the formation of LIG. A comprehensive investigation of the marker inks and of the identified dyes has enabled the formulation of a more refined hypothesis regarding the factors that influence the conversion of certain precursors to LIG. The investigation of selected xanthene dyes (e.g., Eosin Y, Eosin B, Rhodamine 6G, and Fluorescein) revealed that the aromatic structure of these chemicals and their spectral features (e.g. extinction coefficients) are insufficient to promote laser‐induced pyrolysis. Only Eosin Y and Eosin B show the formation of LIG. A thermogravimetric analysis of the dyes indicates that the thermal stability of dyes plays an important role in laser‐induced pyrolysis. Both Eosin Y and Eosin B exhibited a residual weight above 20% at 800°C in air, different from other xanthene dyes. The relatively high residual weight at high temperatures is what allows the LIG to be formed in the first place. In the case that the residual weight at high temperatures is insufficient, there is an absence of material capable of forming LIG.

Investigations of LIG obtained from pure Eosin Y demonstrated that the precursor thickness plays a critical role in the creation of a conductive LIG layer. Although the morphology of LIG from dyes differs from that of LIG from other precursors, other properties such as crystallinity (observed via Raman spectroscopy) or the conductivity (21 ± 9 S cm^−1^) are comparable with LIG from polyimide. High‐magnification SEM images revealed the presence of nanoparticles on the LIG surface, which were identified as NaBr crystals via EDX, XPS, and XRD analysis.

A dispersion of dye and acrylic binder was employed as another precursor for LIG. This enabled the examination of the impact of dye concentration on the formation of LIG. The use of low concentrations of dye (0.26 mol L^−1^) and low power settings resulted in the formation of LIG with a sheet resistance exceeding *R*_s_ > 1000 Ω/□. A high concentration of dye (0.75 mol L^−1^) and high power settings yielded LIG with conductivity comparable to that of LIG from polyimide (34 ± 20 S cm^−1^). LIG from acrylic paint exhibited a porous morphology. As in the case of the pure dye, NaBr crystals were found on the LIG surface.

The use of Eosin dyes in the form of solution or dispersion in various media (like inks and paints) enables the coating of any wettable surface. We proposed a new “Paint & Scribe” methodology for integrating LIG electrodes and conductive tracks onto target surfaces which are unsuitable for direct laser pyrolysis. Some proof of concept demonstrators of “Paint & Scribe” methodology were realized using the commercial marker ink: a LIG temperature sensor on a spray‐coated mug, and the integration of LIG with printed and flexible electronics. This opens up new possibilities in this field, as the tunable and specific properties of LIG are advantageous in certain applications, such as physical and chemical sensing due to its high surface area. Furthermore, a dispersion of dye and acrylic binder could be exploited for creating freestanding LIG. The patterned LIG is easily transferred from a water bath after dissolving the precursors. This unfolds a plethora of avenues for transferring LIG tracks and electrodes to other surfaces or embedding them into crosslinkable polymers or other materials for use in applications. Although some other approaches were presented recently for the obtainment of free‐standing LIG, we deem that the use of a more cost‐effective and customizable precursor like the dye can facilitate a more precise and controlled method of creating and transferring freestanding LIG onto target substrates. This can be advantageous in applications such as stretchable conductors for wearables, soft actuators and soft robotics, as LIG is a promising material for these applications.

## 4 Experimental Section

### Chemicals

A set of non‐permanent markers from Staedtler (Lumocolor non‐permanent pen 315) was used for preliminary tests of laser scribing. Pure dyes powders were then tested: Eosin B (Acros Organics, CAS number 548‐24‐3), Eosin Y (Thermo Fisher Scientific, CAS number 17372‐87‐1), Fluorescein (Sigma‐Aldrich, CAS number 2321‐07‐5), Rhodamine B (Sigma‐Aldrich, CAS number 81‐88‐9), Rhodamine 6G (Sigma‐Aldrich, CAS number 989‐38‐8), Brilliant Blue FCF (TCI America, CAS number 3844‐45‐9), Crystal Violet (Thermo Fisher Scientific, CAS number 548‐62‐9). They were used as received or diluted in water purified by a Millipore Milli‐Q UV system (resistivity >18 MΩ cm).

Dye films were produced by spin coating (10 rps @ 20s) a solution of the dye in methanol onto microscope glass slides (25 × 75 × 1 mm, ISO 8037/1, Epredia). The concentration was 0.3 mol L^−1^ for the laser fluence investigations done by continuous defocusing as shown in Figures 1 and 2.

For the investigation of LIG from pure Eosin Y films, the dye was mixed with methanol and spin coated (10 rps @ 20s) with different concentrations onto microscope glass slides. The nomenclature M2 stands for 0.15 mol L^−1^, M4 for 0.29 mol L^−1,^ and M6 for 0.43 mol L^−1^ of Eosin Y in methanol.

For the investigation of LIG from Eosin Y and acrylic binder mixtures of acrylic binder (Gerstaecker acrylic binder, Ref.Nr.: 20992), Eosin Y and deionized water were prepared at different dye concentration. For each concentration 0.5g of acrylic binder and 0.1g of deionized water were added and manually mixed with the following Eosin Y amounts: AH1 0.1g (0.15 mmol), AH2 0.2g (0.29 mmol), and AH3 0.3g (0.43 mmol). The resulting Eosin Y concentrations of the mixtures were the following: AH1 stands for 0.26 mol L^−1^, AH2 for 0.5 mol L^−1^, and AH3 for 0.75 mol L^−1^. The mixtures were spin‐coated (20 rps @ 20s) onto microscope glass slides.

Acrylic binder (Gerstaecker acrylic binder, Ref.Nr.: 20992 and Aqua‐Cryl CFB by ADLER‐Werk Lackfabrik Johann Berghofer GmbH & Co KG) were coated by hand onto glass slides to investigate their behavior upon laser irradiation.

### Laser Scribing Parameters

A 10.6 µm IR CO_2_ laser cutter/engraver (Universal Laser Systems VLS 2.30, power *P*_max_ = 30 W) equipped with an HPDFO beam collimator (nominal beam size: 30 µm) and a 355 nm UV laser marker system (Keyence MD‐U1000C, *P*_max_ = 3 W) equipped with a digital galvo scanner were used for laser‐induced pyrolysis.

Standard settings for the IR laser were the following: power P = 10%, speed S = 10% (corresponding toto ≈ 100 mm s^−1^), raster resolution of 500 PPI, an image density of 5 (arbitrary scale, defining a spacing between consecutive raster lines of 280 µm). For the screening, three different powers (5%, 10% and 15%) were used, and indicated as PX by the sample naming (for example M6P10D2.1: M6 stands for the ink concentration and type, while P10 stands for P = 10%, and the defocus is indicated by the D2.1 standing for a defocus of d = 2.1 mm.

The settings for the UV laser were the following: power P = 80%, speed S = 200 mm s^−1^, pulse frequency PF = 300 kHz, spot variable SV = ‐50 (basically a defocus setting) and a fill interval FI = 0.015 mm for the scribing. The scribing process was set to unidirectional.

For the continuous defocus investigations, the flat samples supported on glass slides were mounted on a 3D printed wedge with a slope of 10° as described elsewhere.^[^ ^44^ ^]^ The laser was focused on the top portion of the wedge resulting in a defocusing by moving to the thinner side of the wedge (Figure 1c).

The pure acrylic binders were irradiated on the wedge with the same IR laser settings and different powers (P = 10% and 20%).

### Dyes Characterization

UV–vis absorption spectra of ink from commercial markers and of pure dyes were obtained with a Cary 3500 UV–vis spectrophotometer (Agilent), operating in the range of λ = 200–800 nm (spectral band width = 0.5 nm), at 25°C. The analyses were performed on diluted solutions, using transparent quartz cuvettes with an optical path of 1 cm, and distilled water as reference. A solution of the commercial marker inks and dye powders with water were created for the analysis. Samples were analyzed in triplicate. UV–vis spectroscopy was also carried out to obtain the molar absorption coefficient of pure dyes at *λ* = 355 nm (relevant as it is the emission wavelength of the UV laser scribing system), by measuring the absorbance of colored solutions at different concentrations. The slope of the best linear fit of absorbance against concentration was used to calculate the absorption coefficient at a molar concentration of 10^−4^, according to Lambert – Beer's law (diluted solutions).

Attenuated Total Reflection Fourier Transformation Infrared Spectroscopy (ATR‐FTIR) measurements were carried out using a Thermo Nicolet Nexus 870 spectrometer equipped with a liquid nitrogen‐cooled HgCdTe detector and a single reflection diamond crystal ATR unit. Spectra were recorded in the range 4000–600 cm^−1^, collecting 128 scans, with a spectral resolution of 2 cm^−1^. The analyses were performed on commercial marker inks by applying the ink on aluminum foils, and on pure dyes in the form of powder.

Confocal Raman Microscopy was performed with a Renishaw InViaTM Qontor confocal microRaman system, equipped with a front‐illuminated CCD camera and a research‐grade Leica DM 2700 microscope. Spectra were collected using a 50X objective (WD 8.20 mm, NA 0.50) and a 532 nm laser (50 mV maximum power and 1800 l/mm grating), with an exposure time of 1s and 10 accumulations, in a spectral range of 1000–3000 cm^−1^. The analyses were performed on commercial markers by applying the color on silicon crystal, and on pure dyes in the form of powders.

The thermal behavior of pure dyes was investigated through Thermogravimetric Analysis (TGA) using an SDT Q650 TA Instrument, operating between 25 and 1200 °C, at a heating rate of 10 °C min^−1^ in nitrogen flow, or 100 °C min^−1^ in air (gas flow rate of 100 mL min^−1^). The dyes’ thermal behavior was tested under different thermal settings trying to reproduce real conditions that can possibly occur during laser scribing. For each measurement, ≈ 5–10 mg of powder were placed inside an alumina pan. For each dye, at least two measurements were repeated.

The thickness of thin films of dyes was measured with an AlphaStep D‐500 Profilometer from KLA‐Tencor. Two scratches down to the substrate were made to act as reference points for the thickness measurement. The thickness of the dye layer was measured between those scratches and averaged over at least 3 different positions and samples.

### LIG Characterization

Images of the samples from the defocus sweep with the 10° wedge were taken with a tabletop scanner (EPSON Perfection V330).

Sheet resistance was measured with a custom 4‐point probe setup consisting of a Keithley 2602B source meter and four linearly arranged measurement tips with a distance of d_probes_ = 1.5 mm. Measurements were carried out on square samples with a length of 10 mm. Values reported are averaged over 9 measurements and at least 3 samples. The sheet resistance values are corrected with c_geom_ = 0.687.^[^ ^66^ ^]^ The conductivity σ of LIG was calculated using the sheet resistance and thickness values obtained on FIB cross‐sections.

(1) $$\sigma =1/\rho =1/\left(Rs\;*\;t\right)$$

A Focused Ion Beam Scanning Electron Microscope (FIB‐SEM) Helios Nano Lab 600i (Thermo Fisher Scientific, Waltham, MA, USA), equipped with a Bruker XFlash Detector 5010 for energy‐dispersive X‐ray spectroscopy (EDX), was used for characterizing the LIG samples.

Cross‐sections of the material were obtained using the gallium beam impinging perpendicularly on to the sample's surface and operating at 30 kV and 21–65 nA. SEM images were acquired with an Everhart–Thornley detector (ETD) working at 5 kV and 43 pA landing current using secondary electrons (SE) detection. For cross‐section imaging, samples were kept at a working distance of 4–4.2 mm and 52° tilt with respect to the electron beam. The measured values of LIG thickness were corrected for the sample tilt 52°.

SEM images were recorded using a FEG‐SEM Gemini scanning electron microscope (Carl Zeiss Microscopy, Germany), working with acceleration potential of 20 kV and working distance of ≈ 6–8 mm. For elemental analysis, an X‐ray detector (EDX) was used.

Confocal Raman Microscopy was performed to collect spectra on different regions of the LIGs surface, using a 50X objective and a 532 nm laser. Single spectra were acquired with a laser power of 5 mV, an exposure time of 10s and 10 accumulations, in a spectral range of 1000–3000 cm^−1^; maps were collected with a laser power of 5 mV, exposure time of 0.1s and 1 accumulation, in a spectral range of 1200–2700 cm^−1^ (step size 1.5 mm). Measurements were processed with the Renishaw software WiRETM, corrected for cosmic rays, baseline, and noise. If a spectrum with shaded area is shown the measurements were averaged over at least 3 positions.

2D maps were collected on each LIG sample of the M and AH series, with a step size of 1.5 µm along x and y axes, for a total area of ≈ 2 mm^2^, and processed with the Renishaw software WiRETM, operating the same corrections on all spectra (cosmic rays, baseline, and noise reduction). The ‘signal to baseline’ values at 1345 cm^−1^ (D band), 1580 cm^−1^ (G band), and 2665 cm^−1^ (2D band) were extracted over the whole mapped area, and then the 1345/1580 and 2665/1580 bands’ ratios (I_D_/I_G_ and I_2D_/I_G_, respectively) were obtained.

XRD measurements were performed in 2Θ = (10 – 60) ° with a Panalytical Empyrean diffractometer (PANalytical, the Netherlands, Cu tube, λ = 1.5418 Å). The peaks for the comparison with the expected materials were generated using the Inorganic Crystal Structure Database (ICSD), PowderCell and DIFFRAC.EVA.

X‐ray photoelectron spectroscopy (XPS) measurements were performed in an ultrahigh vacuum (UHV) chamber equipped with a dual anode X‐ray source (Al/Mg) and a hemispherical electron energy analyzer (SPECS Phoibos 150). A magnesium X‐ray source with a power of 300W (20mAx15KV) was used.

The TGA of LIG was done by using an SDT Q650 TA Instrument, operating between 25 and 1000 °C, at a heating rate of 10 °C min^−1^ in air flow (60 ml min^−1^). The LIG was scratched from the sample surfaces and collected. In the case of LIG from acrylic binder and dye (AH3) the collected LIG was washed with acetone and deionized water to remove acrylic binder and dye residues. After washing the solution with LIG was filtered, dried and measured.

### Applications

For the temperature sensor demonstrator of “Paint & Scribe” approach a ceramic mug was purchased from a local shop and manually painted with red Staedtler Lumocolor ink by means of an airbrush (Valex) to have a homogeneous coverage. A LIG resistor was scribed on the outer mug surface (laser setting Power = 80%, Speed = 200 mm s^−1^, Pulse frequency = 300 kHz, Spot variable = ‐50, Fill‐Interval = 0.015) to be used as a thermistor. To electrically connect the sensor to the acquisition system, flat connectors were obtained by cutting two stripes (≈ 2 mm wide, 5 cm long) from a 50 µm thick polyimide sheet (Kapton, DUPONT) painted with a conductive Ag paste (CI‐1036, Murabu). A drop of Ag paste was deposited at the thermistor extremities, the flat connectors ends were aligned and placed on them, and the mug was placed in the oven at 120°C for 15 min to allow the Ag to cure and bond the connectors. The sensor was connected to a source measurement unit (Keythley 2604B) and its resistance was measured in a 2‐probe configuration with a current input I_source_ = 1 mA. While filling the mug with cold or hot water, the resistance data were collected and sent to a PC through a Python script. The mug temperature was monitored by taking pictures with a thermal camera (FLIR C3‐X).

For the “Paint & Scribe” approach for the integration with flexible circuit fabrication, a commercial highlighter marker was emptied, refilled with Staedtler Lumocolor® ink, and used to manually draw a line on a flexible Mylar (PET) sheet (50 µm thick). A rectangle of LIG (5 × 10 mm) was obtained from the drawn line by scribing with UV laser (laser setting: Power = 55%, Speed = 200 mm s^−1^, Pulse frequency = 300 kHz, Spot variable = ‐50, Fill‐Interval = 0.015). The unconverted remaining/extra red dye was dissolved and removed by dipping the sample in water for a few seconds. Two lateral silver tracks were patterned at the LIG element sides by manually depositing a screen printable Ag paste (CI‐1036, Murabu) with the help of a plastic mask. The Ag paste was cured in an oven at 100°C for 15 min. The demonstrative flexible circuital element resistance was measured with multimeter probes connected to the silver tracks.

For the demonstrators with LIG from acrylic binder the sample AH3 with a concentration 0.75 mol L^−1^ of Eosin Y was used as described before. The LIG patterns were scribed with the IR CO_2_ laser cutter with the following settings: P = 10%, S = 10%, PPI = 500, ID = 5, d = 2.2 mm. After the transfer of the LIG onto a glass substrate (25 × 75 × 1 mm, ISO 8037/1, Epredia) the temperature sensor was contacted with silver conductive paint (Leitsilber 200) and electrical connection legs for ITO glass substrates. The resistance of the temperature sensor was measured with a Keithley 2601B source meter sourcing 1 mA. The temperature was changed by placing the sample onto a heating plate at T = 80°C. The stretchable conductor was made by embedding the freestanding LIG into PDMS (SYLGARD 184), manually mixed at a 10:1 ratio and degassed. A glass substrate (25 × 75 × 1 mm, ISO 8037/1, Epredia) was coated with a thin layer of PDMS by hand and the freestanding LIG was placed onto this layer, a small amount of PMDS was used to cover the placed LIG with another layer to obtain full embedding of the LIG track. The sample was cured on a heating plate at 100°C until completely crosslinked, cut in form with a scalpel blade and peeled from the glass substrate.

Characterization of commercial Lumocolor**^©^** non‐permanent marker inks using UV–vis, ATR‐FTIR and Raman spectroscopy and identification of used dyes.

Additional data and images including: UV–vis, Raman, EDX, and XPS spectra of dyes and LIG, SEM images (and FIB cross sections) of LIG, and scanned images of UV laser scribed dyes. Additional tables with data reported for corresponding EDX and XPS spectra. Photographs of the PPS approach of LIG integration with printed and flexible electronics.

## Conflict of Interest

The authors declare no conflict of interest.

## Supporting information



Supporting Information

## Data Availability

The data that support the findings of this study are available from the corresponding author upon reasonable request.
