# Supplementary material for: Laser‐Induced Graphene from Commercial Inks and Dyes
Source: Adv Sci (Weinh). 2025 Feb 14;12(14):2412167. doi: 10.1002/advs.202412167 (PMC11984850; doi:10.1002/advs.202412167)
Supplement: Supplementary file 1 — Supporting Information [file ADVS-12-2412167-s001.pdf]

ADVANCED SCIENCE Open Access

Supporting Information

for *Adv. Sci.*, DOI 10.1002/advs.202412167

Laser-Induced Graphene from Commercial Inks and Dyes

*Alexander Dallinger, Rachel Camerini, Paola Parlanti, Mauro Gemmi, Sreenadh Thaikkattu Sankaran, Marina Galliani, Birgit Kunert, Rodorico Giorgi and Francesco Greco**

Supporting Information

Laser-Induced Graphene from Commercial Inks and Dyes

Alexander Dallinger[1], Rachel Camerini[2], Paola Parlanti[3], Mauro Gemmi[3], Sreenadh Thaikkattu Sankaran[4,5], Marina Galliani[4,5], Birgit Kunert[1], Rodorico Giorgi[2], Francesco Greco[1,4,5,6,*]

1 Institute of Solid State Physics, NAWI Graz, Graz University of Technology, 8010 Graz, Austria.

2 Center for Colloid and Surface Science - CSGI and Department of Chemistry, University of Florence, via della Lastruccia 3-50019, Sesto Fiorentino, Italy

3 Center for Materials Interfaces, Electron Crystallography, Istituto Italiano di Tecnologia, Viale Rinaldo Piaggio 34, 56025 Pontedera, Italy

4 The Biorobotics Institute, Scuola Superiore Sant'Anna, Viale Rinaldo Piaggio 34, 56025 Pontedera, Italy.

5 Department of Excellence in Robotics & AI, Scuola Superiore Sant'Anna, Piazza Martiri della Libertà 33, 56127 Pisa, Italy.

6 Interdisciplinary Center on Sustainability and Climate, Scuola Superiore Sant'Anna, Piazza Martiri della Libertà 33, 56127 Pisa, Italy.

E-mail: Francesco.Greco@santannapisa.it

Characterization of dyes in the commercial markers

This section explains in detail the identification and characterization of the commercial Lumocolor® non-permanent marker inks using UV-Vis, ATR-FTIR and Raman spectroscopy.
The red Lumocolor® marker showed characteristics of Eosin Y in all three spectra. The UV-Vis spectra (Figure S0.1a) of Eosin Y [1] and the red Lumocolor® marker shows the same features and a maximum at λ = 517 nm. The FTIR spectra (Figure S0.1b) of the red Lumocolor® marker (transmission) and the reference Eosin Y spectra (absorption) [2] show comparable peak features. Also the Raman spectra (Figure S0.1c) of the red Lumocolor® marker and the reference Eosin Y spectra [3] are matching which identifies the dye in the red Lumocolor® marker as Eosin Y.

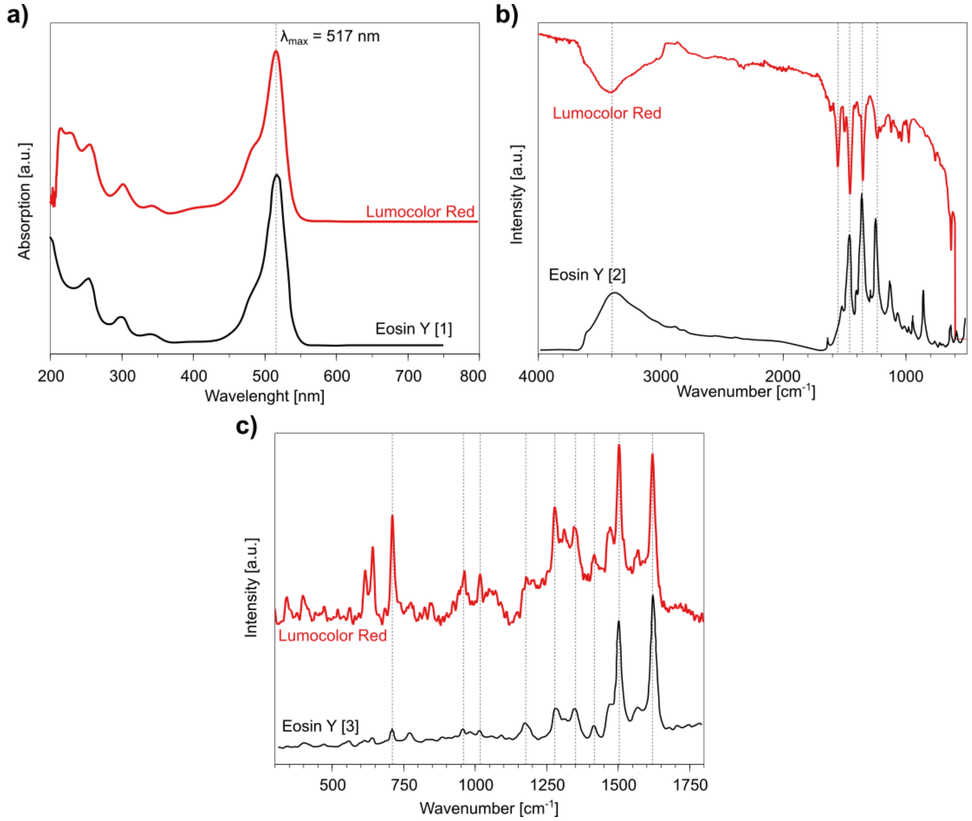

Figure S0.1: Red Lumocolor® dye identification via a) UV-Vis [1], b) FTIR [2] and c) Raman spectroscopy [3] with a Eosin Y from references as noted.

---

[1] Hossain, Ashraf, A. B. M. Sadique Rayhan, Md. Jahir Raihan, Aklima Nargis, Iqbal M. I. Ismail, Ahsan Habib, and Abu Jafar Mahmood. „Kinetics of Degradation of Eosin Y by One of the Advanced Oxidation Processes (AOPs)—Fenton's Process“. *American Journal of Analytical Chemistry* 07, Nr. 12 (2016): 863–79. https://doi.org/10.4236/ajac.2016.712074.

[2] Sabatini, Francesca, Eva Eis, Ilaria Degano, Mathieu Thoury, Ilaria Bonaduce, and Anna Lluveras-Tenorio. „The Issue of Eosin Fading: A Combined Spectroscopic and Mass Spectrometric Approach Applied to Historical Lakes“. *Dyes and Pigments* 180 (September 2020): 108436. https://doi.org/10.1016/j.dyepig.2020.108436.

[3] Saviello, Daniela, Alexandra Di Gioia, Pierre-Ives Turenne, Maddalena Trabace, Rodorico Giorgi, Antonio Mirabile, Piero Baglioni, and Daniela Iacopino. „Handheld Surface-enhanced Raman Scattering Identification of Dye Chemical Composition in Felt-tip Pen Drawings“. *Journal of Raman Spectroscopy* 50, Nr. 2 (February 2019): 222–31. https://doi.org/10.1002/jrs.5411.

The purple Lumocolor® marker exhibited characteristics analogous to those of the red Lumocolor® marker (Eosin Y) and Crystal Violet. The UV-Vis spectra (Figure S0.2a) of Crystal Violet [4] and the purple Lumocolor® marker exhibits comparable features, as well as peaks analogous to those observed in the red Lumocolor® marker. The FTIR spectra (Figure S0.2b) of the purple Lumocolor® marker (transmission) and the reference Crystal Violet spectra (transmission) [5] exhibit comparable peak features, as well as peaks that are similar to those of the red Lumocolor® marker. Furthermore, the Raman spectra (Figure S0.2c) of the purple Lumocolor® marker and the reference Crystal Violet spectra [6] exhibit a similar pattern, yet also display peaks that are comparable to those of the red Lumocolor® marker. This indicates that the dye in the purple Lumocolor® marker is a mixture of Crystal Violet and Eosin Y.

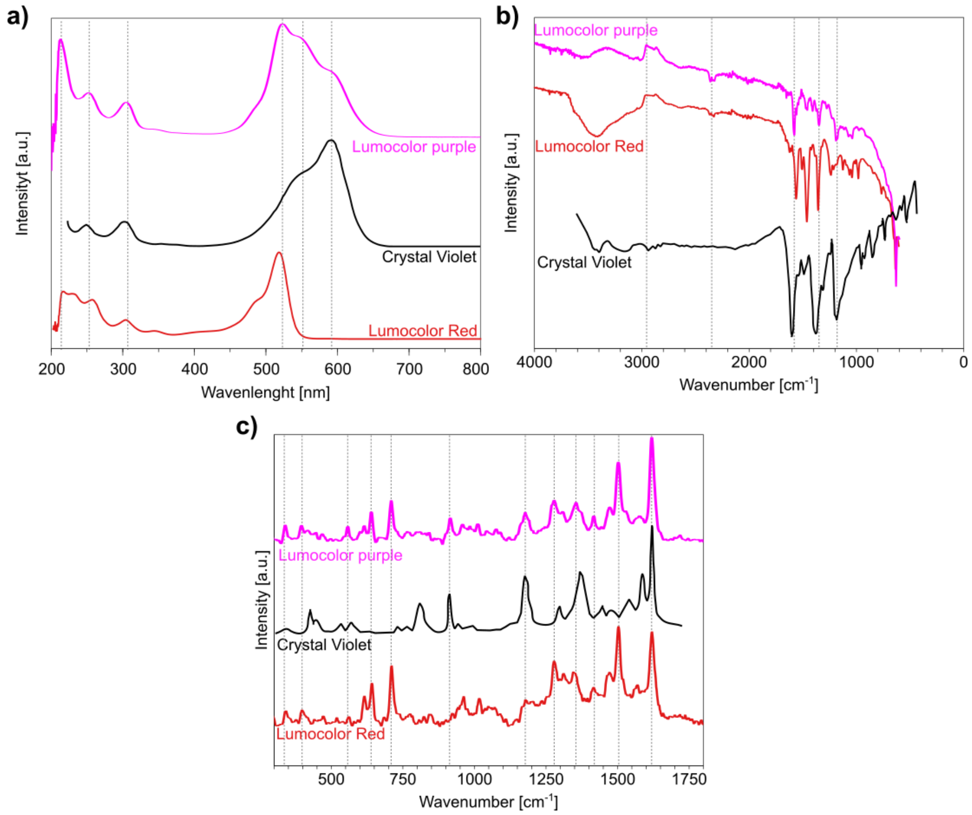

Figure S0.2: Purple Lumocolor® dye identification via a) UV-Vis [4], b) FTIR [5] and c) Raman spectroscopy [6] with a Crystal Violet spectra from references as noted.

[4] Rehman, Faiza, Sayed Murtaza, Javed Ali Khan, and Hasan M Khan. „REMOVAL OF CRYSTAL VIOLET DYE FROM AQUEOUS SOLUTION BY GAMMA IRRADIATION". Journal of the Chilean Chemical Society 62, Nr. 1 (March 2017): 3359–64. https://doi.org/10.4067/S0717-97072017000100011.
[5] Abdi, Mastaneh, Mona Balagabri, Hazhir Karimi, Hooshyar Hossini, and Seyed Omid Rastegar. „Degradation of Crystal Violet (CV) from Aqueous Solutions Using Ozone, Peroxone, Electroperoxone, and Electrolysis Processes: A Comparison Study". *Applied Water Science* 10, Nr. 7 (Juli 2020): 168. https://doi.org/10.1007/s13201-020-01252-w.
[6] Cesaratto, Anna, Marco Leona, and Federica Pozzi. „Recent Advances on the Analysis of Polychrome Works of Art: SERS of Synthetic Colorants and Their Mixtures With Natural Dyes". *Frontiers in Chemistry* 7 (4. March 2019): 105. https://doi.org/10.3389/fchem.2019.00105.

The blue Lumocolor® marker exhibited characteristics of Brilliant Blue FCF. The UV-Vis spectra (Figure S0.3a) of Brilliant Blue FCF [7] and the blue Lumocolor® marker exhibits comparable features with the expectation of a peak 540 nm (absorbed yellow/green, emitted violet). The FTIR spectra (Figure S0.3b) of the blueLumocolor® marker (transmission) and the reference Brilliant Blue spectra (transmission) [8] exhibit comparable peak features. The Raman spectra (Figure S0.3c) of the blue Lumocolor® marker and the reference Brilliant Blue spectra [9] exhibit a similar pattern, which concludes that Brilliant Blue is most likely to be the dye in the blue Lumocolor® marker.

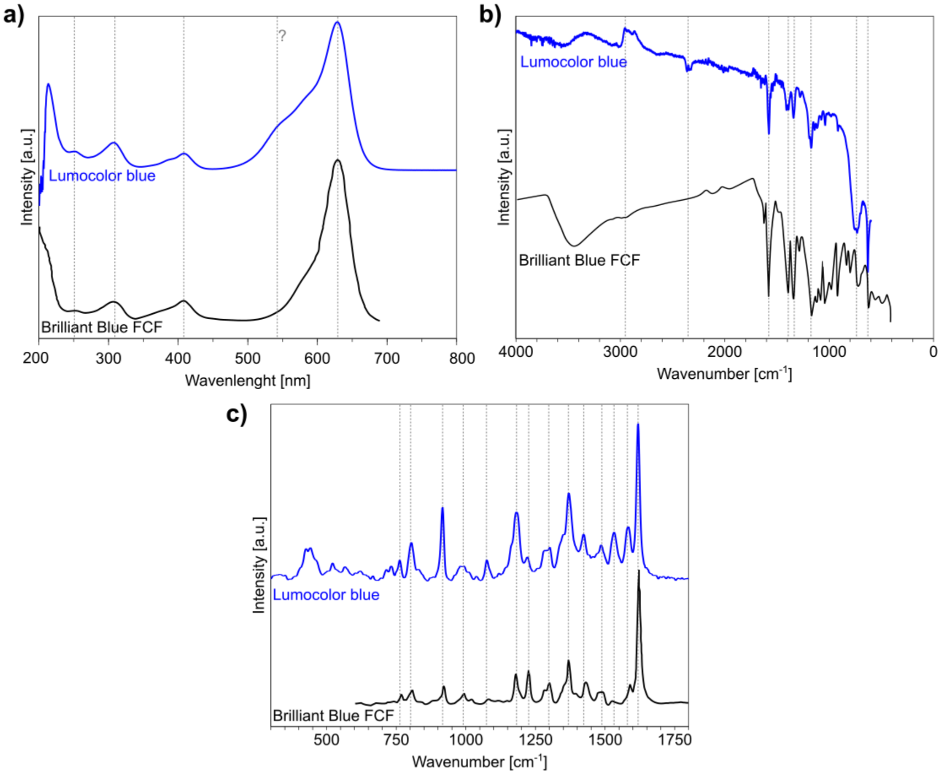

Figure S0.3: Blue Lumocolor® dye identification via a) UV-Vis [7], b) FTIR [8] and c) Raman spectroscopy [9] with a Brilliant Blue FCF from references as noted.

The green Lumocolor® marker exhibited characteristics of Brilliant Blue FCF and of the yellow Lumocolor® marker with an unknown dye.The UV-Vis spectra (Figure S0.4a) of Brilliant Blue

[7] Pavanelli, Sérgio P., Glayson Leonardo Bispo, Clésia Cristina Nascentes, and Rodinei Augusti. “Degradation of Food Dyes by Zero-Valent Metals Exposed to Ultrasonic Irradiation in Water Medium: Optimization and Electrospray Ionization Mass Spectrometry Monitoring.” *Journal of the Brazilian Chemical Society* 22, no. 1 (January 2011): 111–19. https://doi.org/10.1590/S0103-50532011000100015.
[8] Drhimer, Fatine, Maryem Rahmani, Boutaina Regraguy, Souad El Hajjaji, Jamal Mabrouki, Abdeltif Amrane, Florence Fourcade, and Aymen Amine Assadi. “Treatment of a Food Industry Dye, Brilliant Blue, at Low Concentration Using a New Photocatalytic Configuration.” *Sustainability* 15, no. 7 (March 27, 2023): 5788. https://doi.org/10.3390/su15075788.
[9] Chen, Hsiao-Chien, Ching-Hsiang Chen, Chia-Shuo Hsu, Tai-Lung Chen, Mei-Yi Liao, Chia-Ching Wang, Chia-Fen Tsai, and Hao Ming Chen. “In Situ Creation of Surface-Enhanced Raman Scattering Active Au–AuO $_x$ Nanostructures through Electrochemical Process for Pigment Detection.” *ACS Omega* 3, no. 12 (December 31, 2018): 16576–84. https://doi.org/10.1021/acsomega.8b02677.

FCF [7] and the green Lumocolor® marker exhibit comparable features with the expectation of a peak 450 nm (absorbed blue, emitted orange/yellow), which is ascribable to the yellow Lumocolor® marker. The FTIR spectra (Figure S0.4b) of the blueLumocolor® marker (transmission) and the reference Brilliant Blue spectra (transmission) [8] exhibit comparable peak features, with the exception of a signal around 1500 cm^{-1} and at 776 cm^{-1} , which are ascribable to the presence of the components of yellow Lumocolor® marker. The Raman spectra (Figure S0.4c) of the green Lumocolor® marker and the reference Brilliant Blue spectra [9] exhibit a similar pattern, with the exception of two peaks (1145 cm^{-1} and 1400 cm^{-1}) ascribable to the presence of components from yellow Lumocolor® marker. This means that Brilliant Blue is most likely the main dye in the blue Lumocolor® marker with the mixture of the unknown dye from yellow Lumocolor® marker.

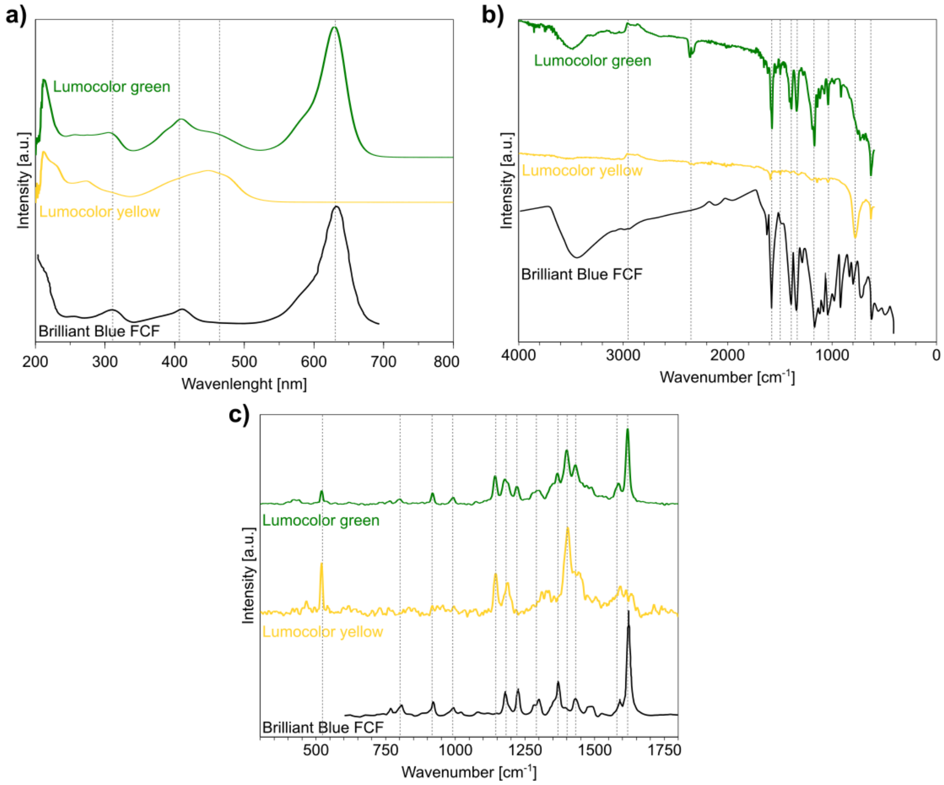

Figure S0.4: Green Lumocolor® dye identification via a) UV-Vis [7], b) FTIR [8] and c) Raman spectroscopy [9] with a Brilliant Blue FCF from references as noted.

The orange and brown Lumocolor® markers have similar spectral patterns (Figure S1 and S2) and are likely a combination of Eosin Y and the unknown dye of the yellow Lumocolor® marker mixed in different proportions.

Additional Figures and Tables

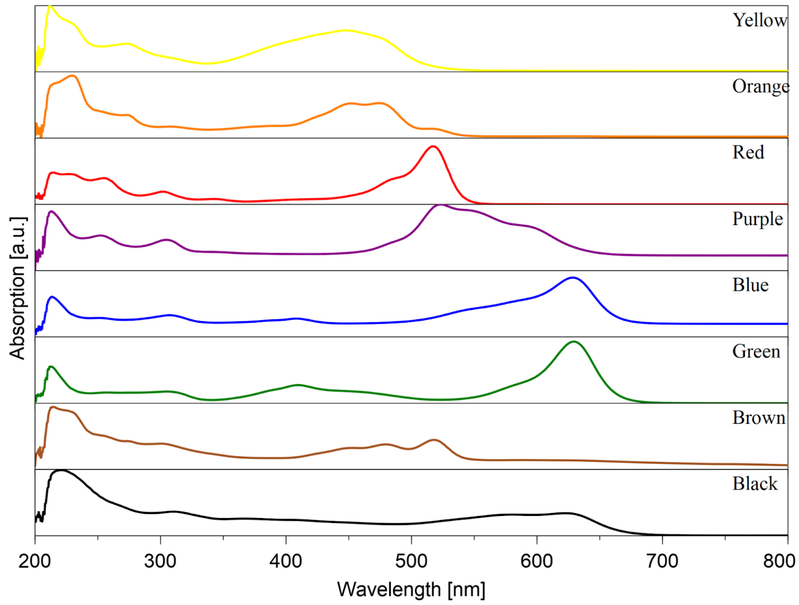

Figures S1: UV-Vis spectra of all 8 Lumocolor® non-permanent marker inks.

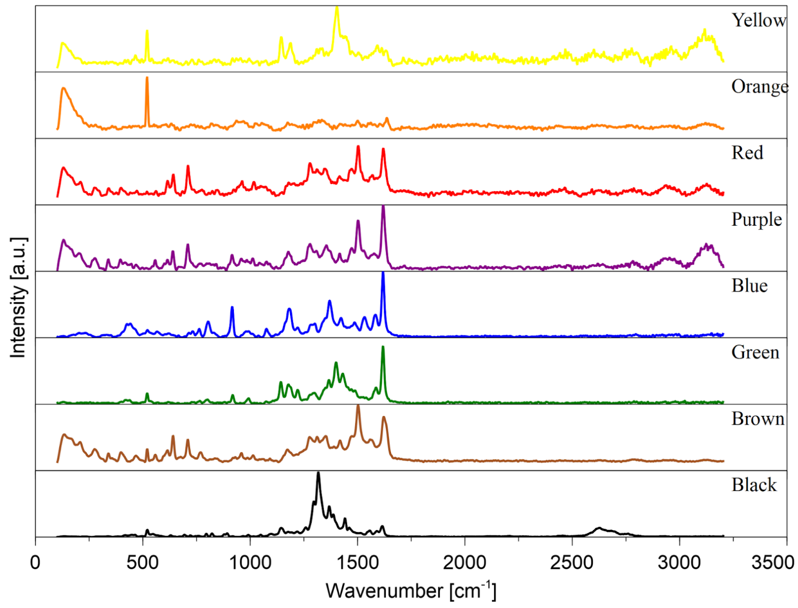

Figure S2: Raman spectroscopy of all the Lumocolor® non-permanent marker inks.

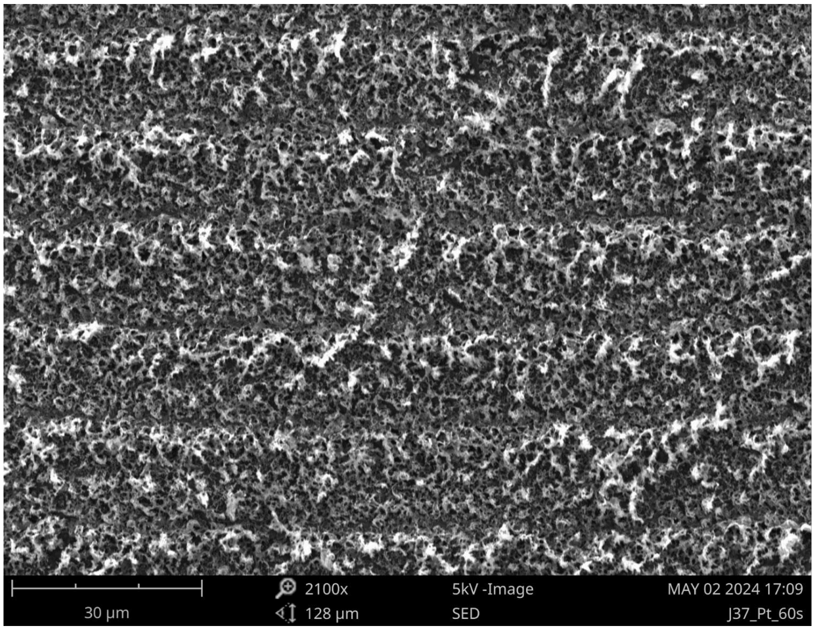

Figure S3: SEM of Brilliant Blue thin film after laser irradiation

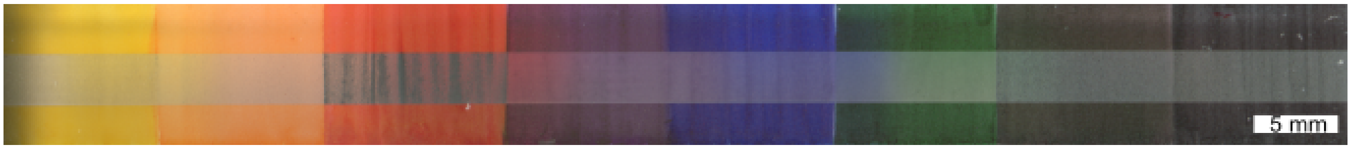

Figure S4: Scanning image of Lumocolor® markers irradiated with an UV laser

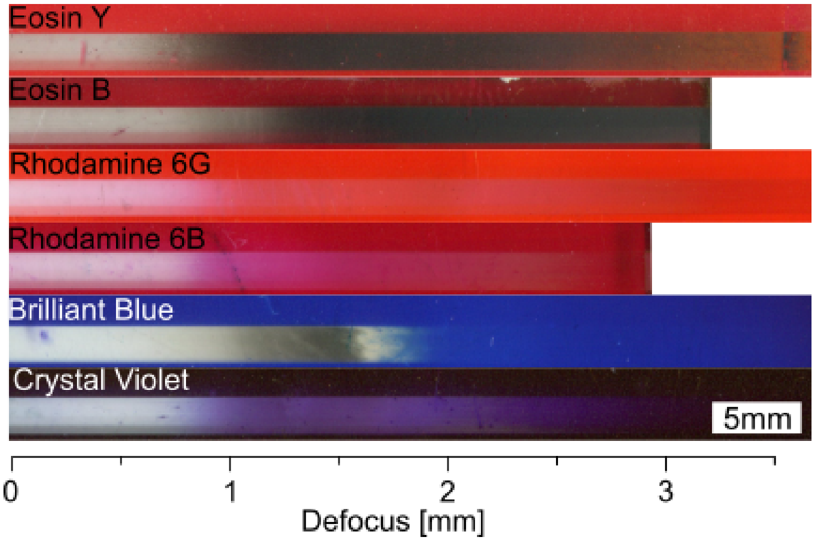

Figure S5: Investigation of selected dyes via continuous defocus irradiated with an UV laser.

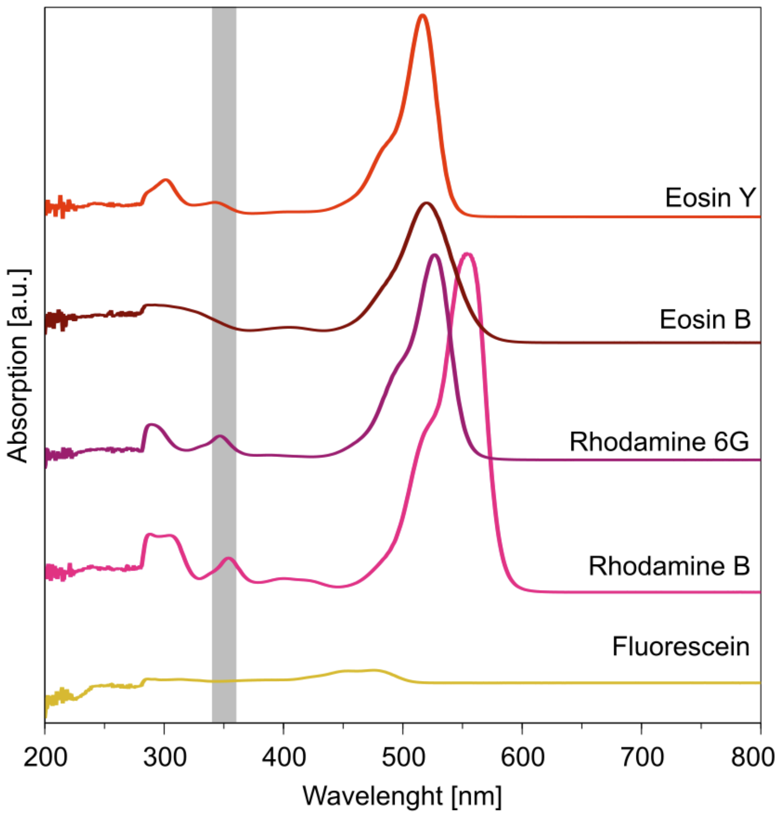

Figure S6: UV/VIS spectra of Xanthene dyes

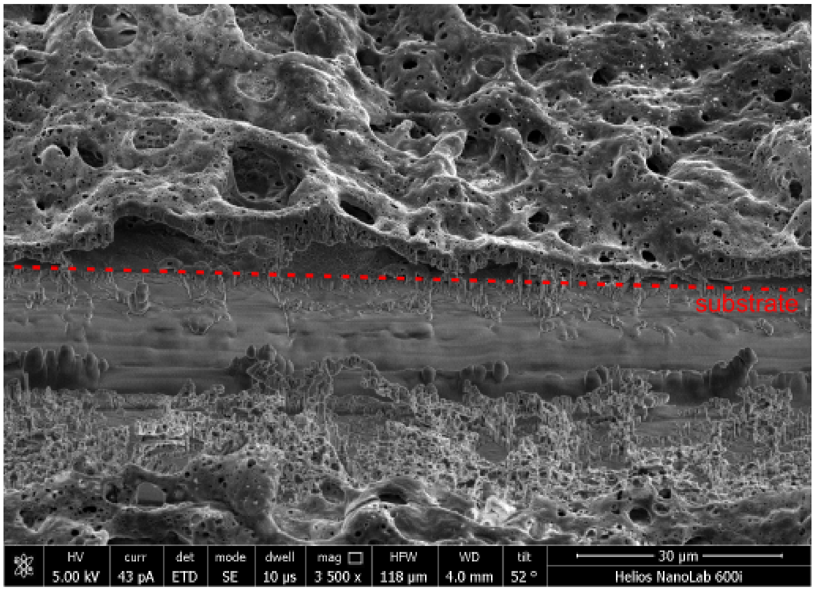

Figure S7: SEM image for the estimation of thickness of LIG obtained from M6P10D2.1 (cross section by FIB).

Table S1: Peak position and the full-width at half-maximum (FWHM) for LIG from M6

Sample	Peak no.	Name	Centre [cm^{-1}]	Width (FWHM) [cm^{-1}]
M6P5D0	1	D	1338.7 ± 2.7	69.7 ± 16.9
	2	G	1582.3 ± 4.5	74.1 ± 12.4
	3	2D	2668.3 ± 5.3	93.7 ± 14.7
M6_P10D2.1	1	D	1337.1 ± 2.1	72.4 ± 10.1
	2	G	1582.8 ± 3.8	74.5 ± 11.4
	3	2D	2662.4 ± 3.0	78.8 ± 36.7
M6_P20D1.9	1	D	1343. ± 1.1	62.4 ± 19.5
	2	G	1579.5 ± 3.6	54.7 ± 15.1
	3	2D	2680.8 ± 3.3	79.8 ± 5.2

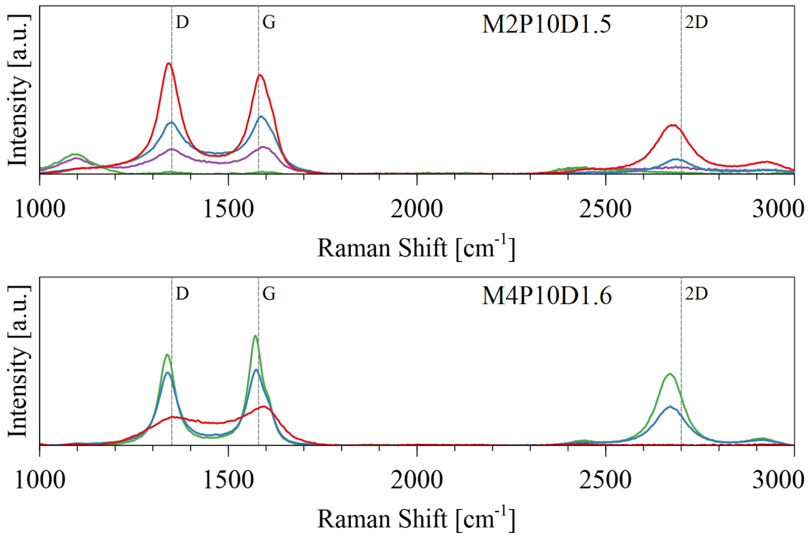

Figure S8: Raman Spectra of carbonized Eosin Y samples M2 and M4

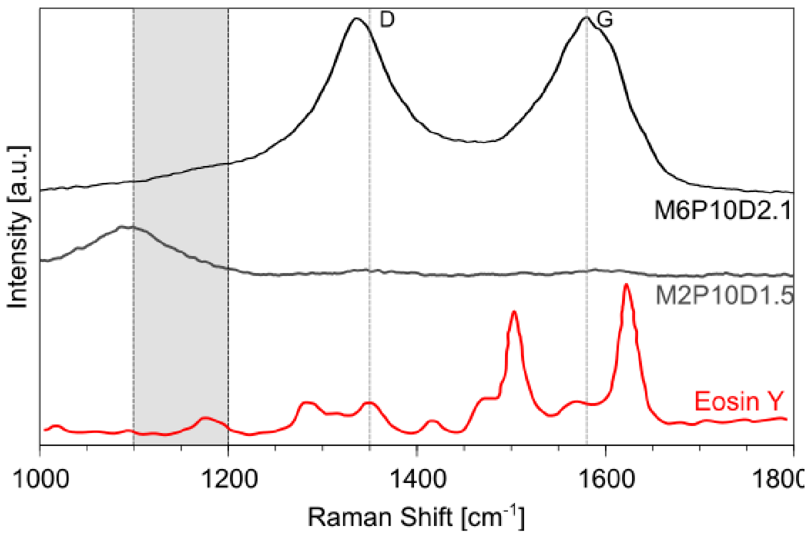

Figures S9: Raman spectra of a sample with low sheet resistance (M6P10D2.1), high sheet resistance (M2P10D1.5) and Eosin Y reference [3].

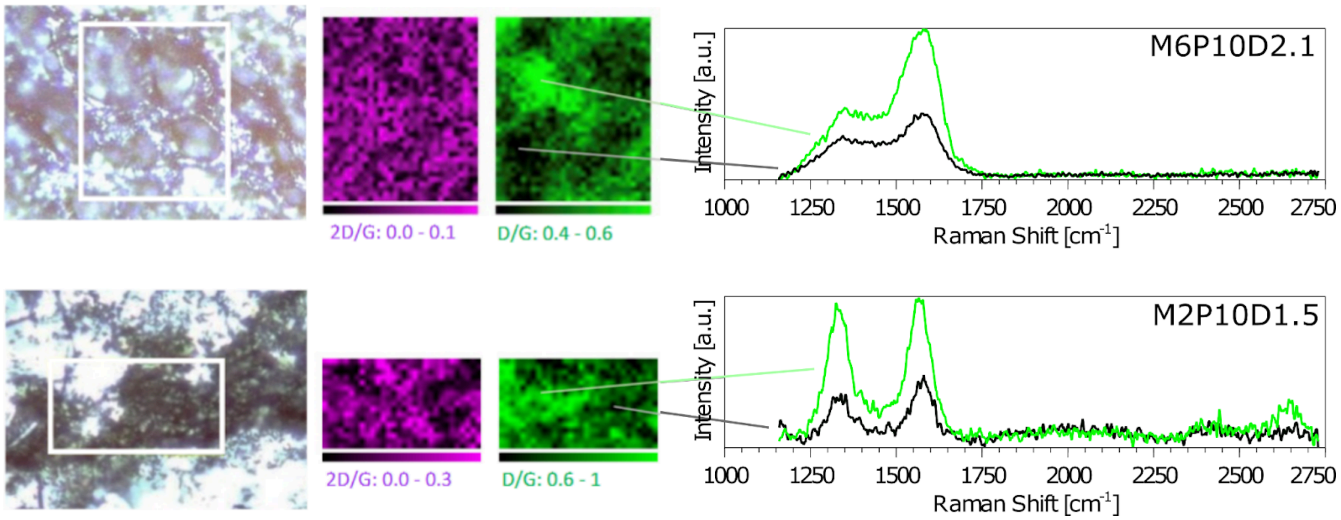

Figure S10: 2D Raman Maps of M2 and M6 samples

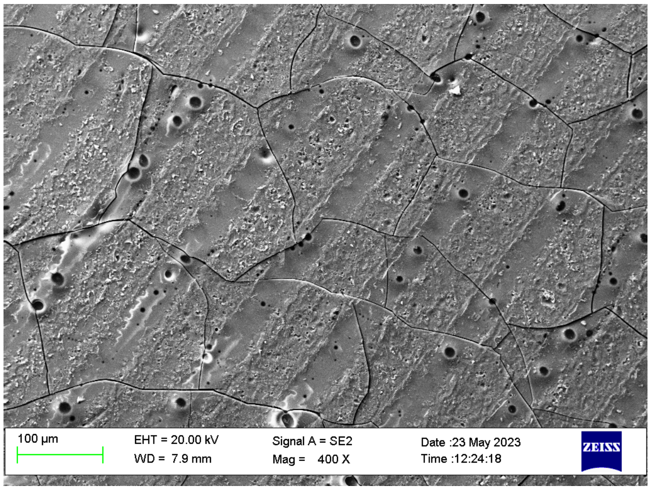

Figures S11: SEM images of M2P10D1.5 after laser irradiation

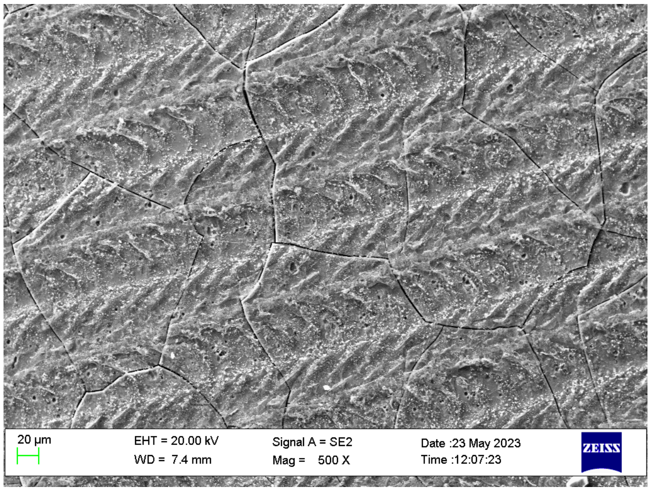

Figures S12: SEM M4P10D1.6 after laser irradiation.

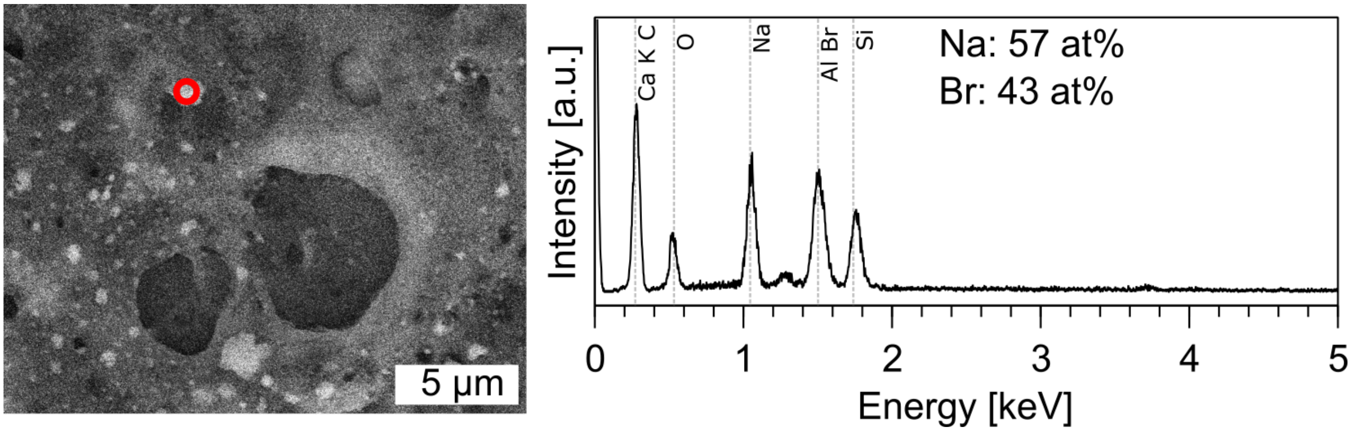

Figure S13: SEM image and EDX of white spot (red circle) on LIG surface on M6P10D2.1 showing a Na and Br ratio of nearly 1:1.

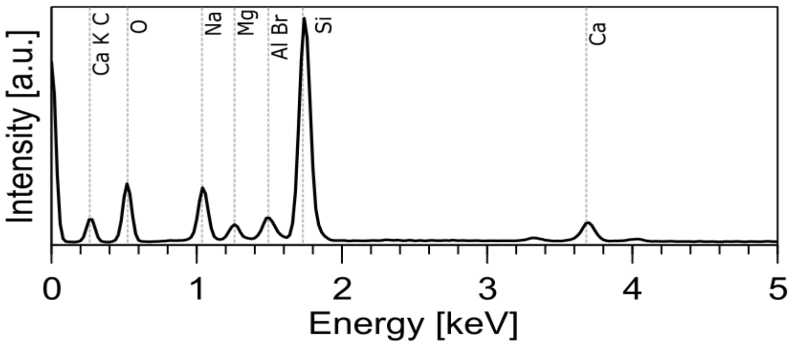

Figure S14: EDX spectrum of M2P5D0.4

Tables S2: Atomic percentages obtained via EDX of M2 and M6 samples

SAMPLE	C K	O K	Na K	Mg K	Al K	Si K	K K	Ca K	Br L
M2P5D0.4	27.78	46.99	4.99	1.42	0.21	16.45	0.23	1.53	0.76
M6P10D2.1	67.88	20.64	4.21	0.35	-0.05	4.43	0.06	0.36	2.11

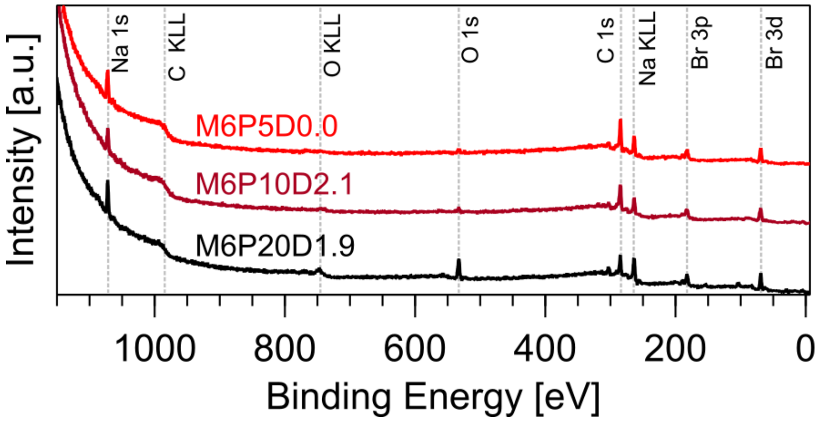

Figure 15: XPS spectra of M6 samples

Table S3: XPS Data of M6 samples

SAMPLE	C 1s (at%)	O 1s (at%)	Na 1s (at%)	Br 3p (at%)
M6P5D0.0	77.6	5.9	10.14	6.35
M6P10D2.1	79.19	6.7	7.61	6.5
M6P20D1.9	52.84	26.58	11.82	8.76

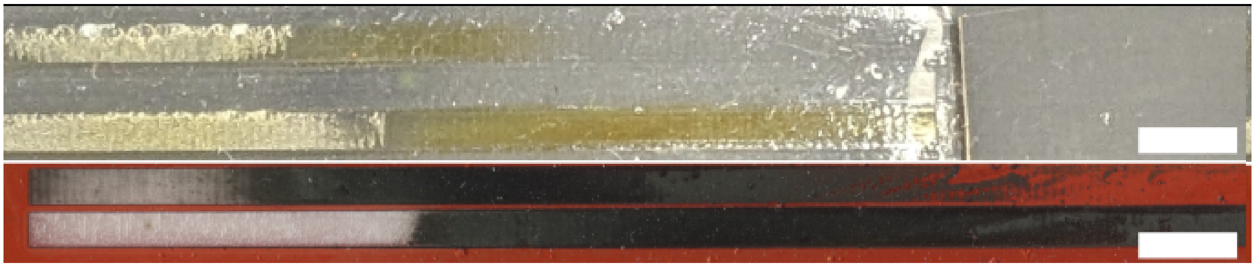

Figure S16: Laser scribed acrylic binder showing no carbonization (top, scale bar = 5 mm), after mixing with Eosin Y (AH3) the material showed carbonization (bottom, scale bar = 5 mm)

Aqua-Cryl CFB (colourless wood paint by ADLER-Werk Lackfabrik Johann Berghofer GmbH & Co KG) showing no sign of carbonization after laser scribing (top, scale bar = 5 mm), after mixing with Eosin Y (same concentration as AH2) the material showed carbonization and had a sheet resistance of around 40 Ω/□ at P= 10% D = 2.2mm settings (bottom, scale bar = 5 mm)

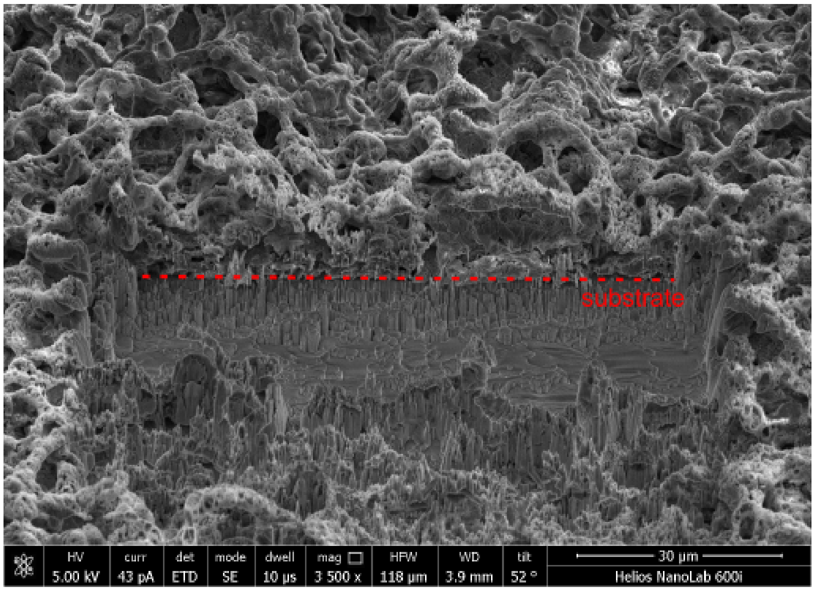

Figure S17: SEM image for the estimation of thickness of LIG obtained from AH3P20D3.6 (cross section by FIB).

Table S4: Peak position and the full-width at half-maximum (FWHM) for LIG from Eosin Y and acrylic binder

Sample	Peak No.	Name	Center [cm^{-1}]	Width (FWHM) [cm^{-1}]
AH2P10D2.1	1	D	1339.2 ± 3.1	66.2 ± 15.8
	2	G	1580.1 ± 8.1	65.5 ± 14.9
	3	2D	2671.2 ± 7.3	82.6 ± 7.8
AH2P20D3.8	1	D	1338. ± 1.7	95.9 ± 6.3
	2	G	1587.3 ± 1.7	79.7 ± 5.9
AH3P10D2.2	1	D	1343.5 ± 3.6	95.9 ± 14.1
	2	G	1464.2 ± 144.	70.2 ± 13.9
	3	2D	2669.9 ± 4.5	89.3 ± 7.7
AH3P20D3.6	1	D	1339.8 ± 0.6	62.4 ± 2.9
	2	G	1582.3 ± 1.0	66.7 ± 0.7
	3	2D	2668.6 ± 3.6	106.2 ± 6.

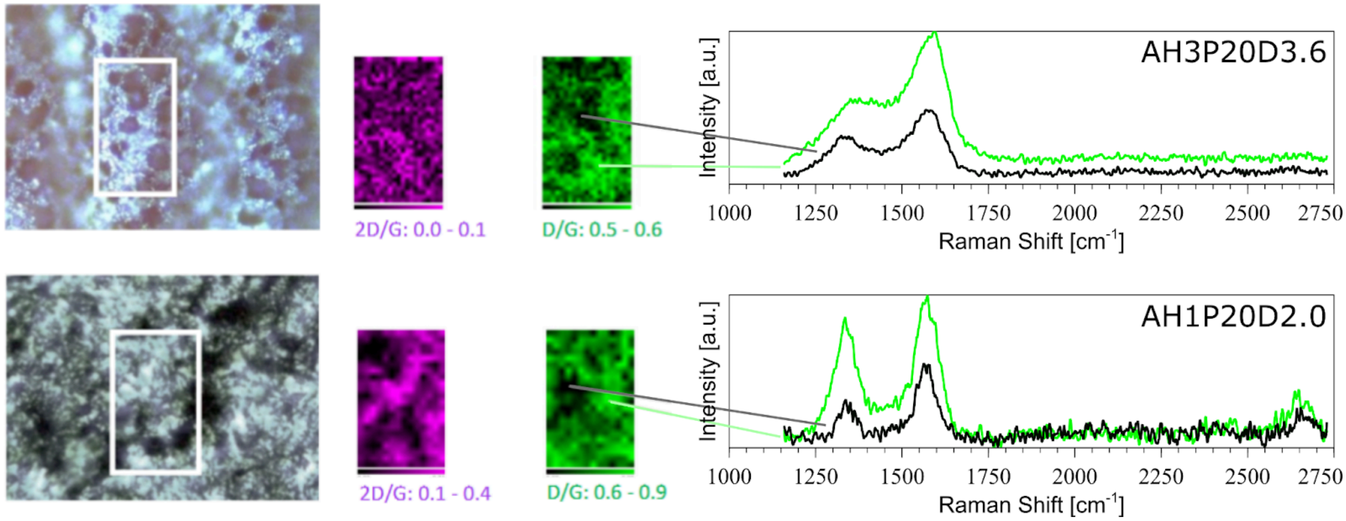

Figure S18: 2D Raman Maps of AH1 and AH3 samples

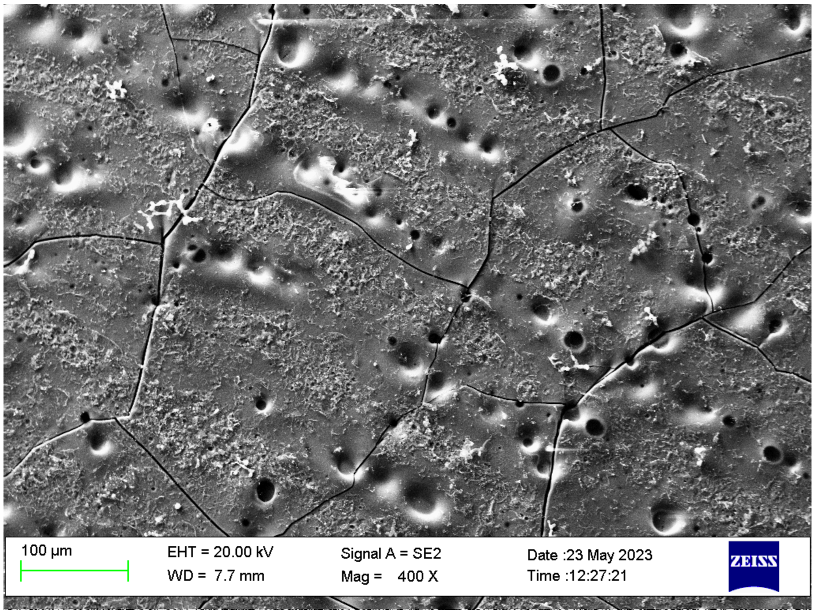

Figure S19: SEM image of AH1 sample

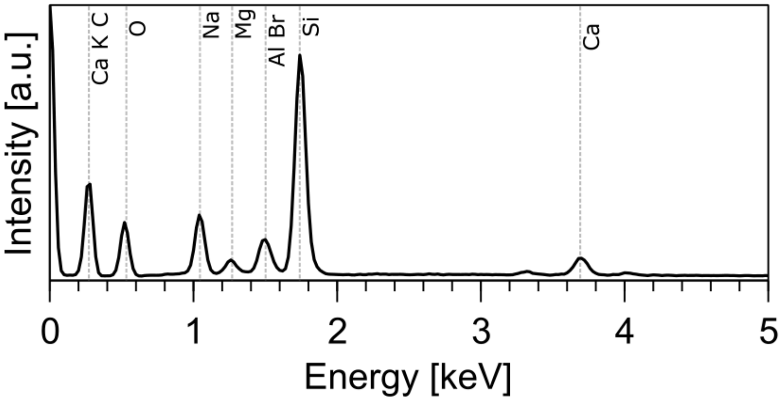

Figure S20: EDX spectrum of AH1P10D2.1

Table S5: Atomic percentages obtained via EDX of AH1 and AH3 samples

SAMPLE	C K	O K	Na K	Mg K	Al K	Si K	Cl K	K K	Ca K	Br L
AH1P10D2.1	60.73	25.78	3.33	0.59	0	7.99	0	0.14	0.79	0.63
AH3P20D3.6	93.44	1.33	1.93	0.08	0.04	1.1	0.05	0.03	0.18	1.82

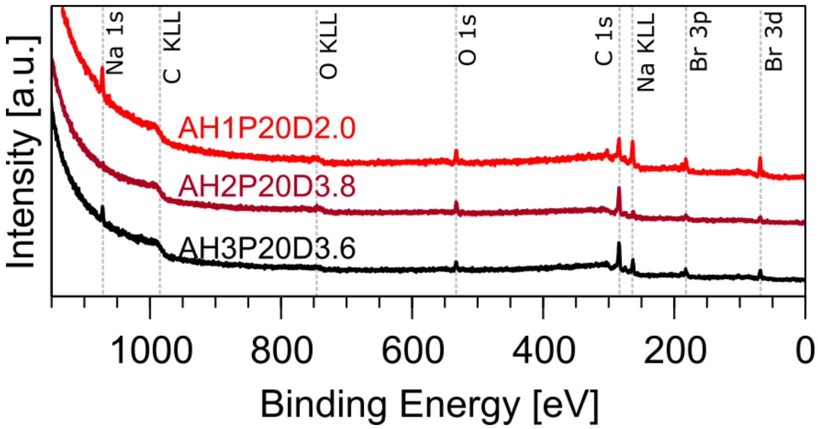

Figure S21: XPS spectrum for AH samples at P = 20%

Table S6: XPS data of AH samples at P = 20%

SAMPLE	C 1s (at%)	O 1s (at%)	Na 1s (at%)	Br 3p (at%)
AH1P20D2.0	64.38	15.83	10.24	9.55
AH2P20D3.8	79.44	14.89	1.28	4.39
AH3P20D3.6	81.4	8.84	4.95	4.82

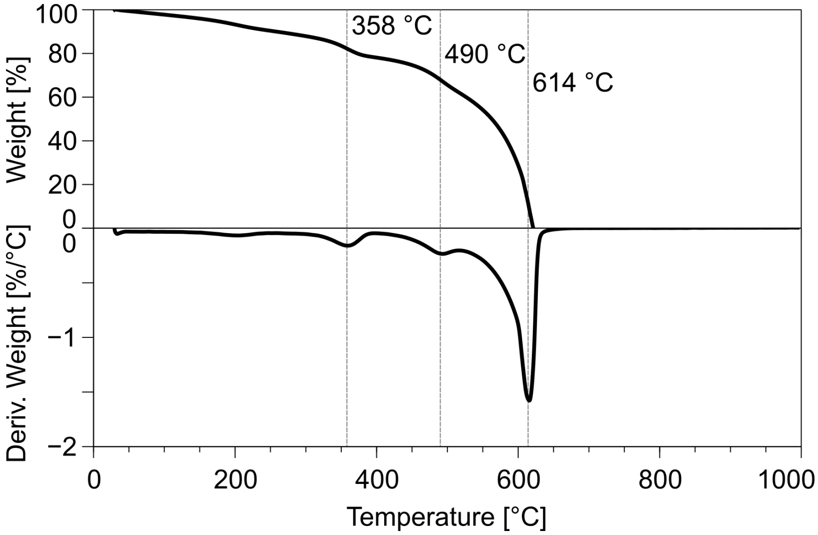

Figure S22: TGA analysis of LIG from AH3

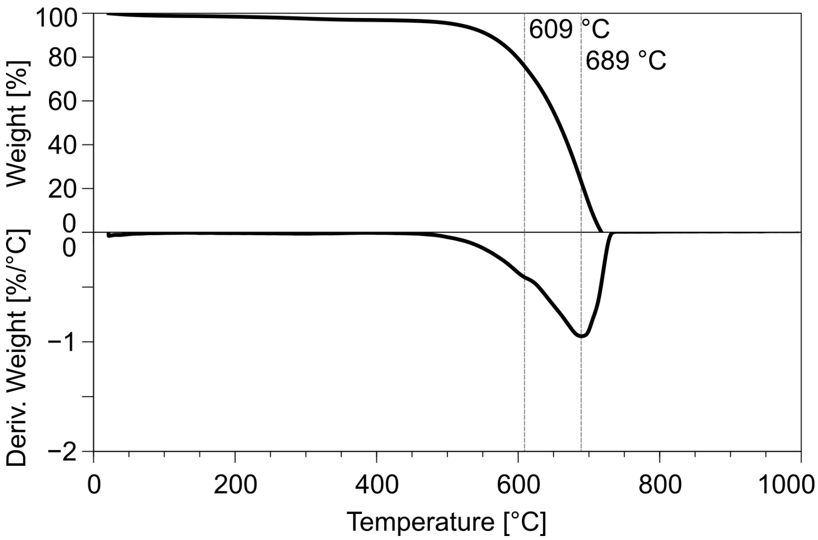

Figure S23: TGA analysis of LIG from Polyimide (see [10] for details on LIG)

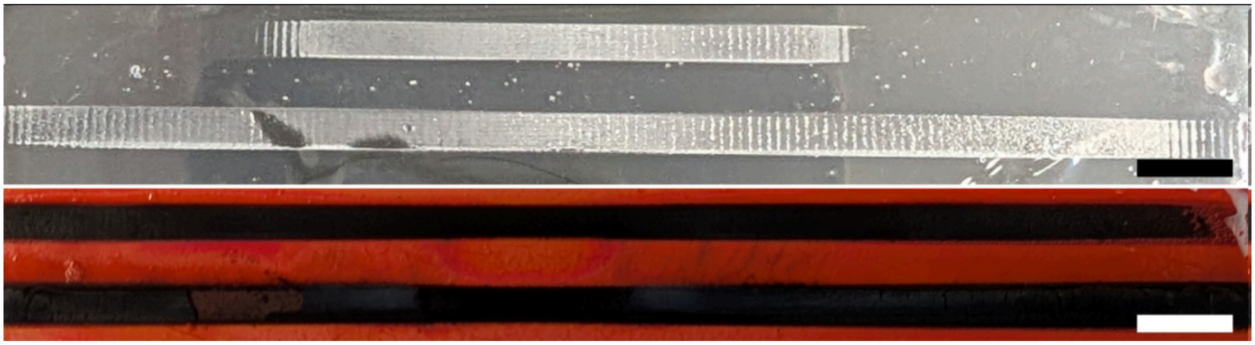

Figure S24: Aqua-Cryl CFB (colourless wood paint by ADLER-Werk Lackfabrik Johann Berghofer GmbH & Co KG) showing no sign of carbonization after laser scribing (top, scale bar = 5 mm), after mixing with Eosin Y (same concentration as AH2) the material showed carbonization and had a sheet resistance of around 40 Ω/□ at P= 10% D = 2.2mm settings (bottom, scale bar = 5 mm)

[10] Dallinger, A.; Keller, K.; Fitzek, H.; Greco, F. Stretchable and Skin-Conformable Conductors Based on Polyurethane/Laser-Induced Graphene. ACS Appl. Mater. Interfaces 2020, 12 (17), 19855–19865. https://doi.org/10.1021/acsami.0c03148.

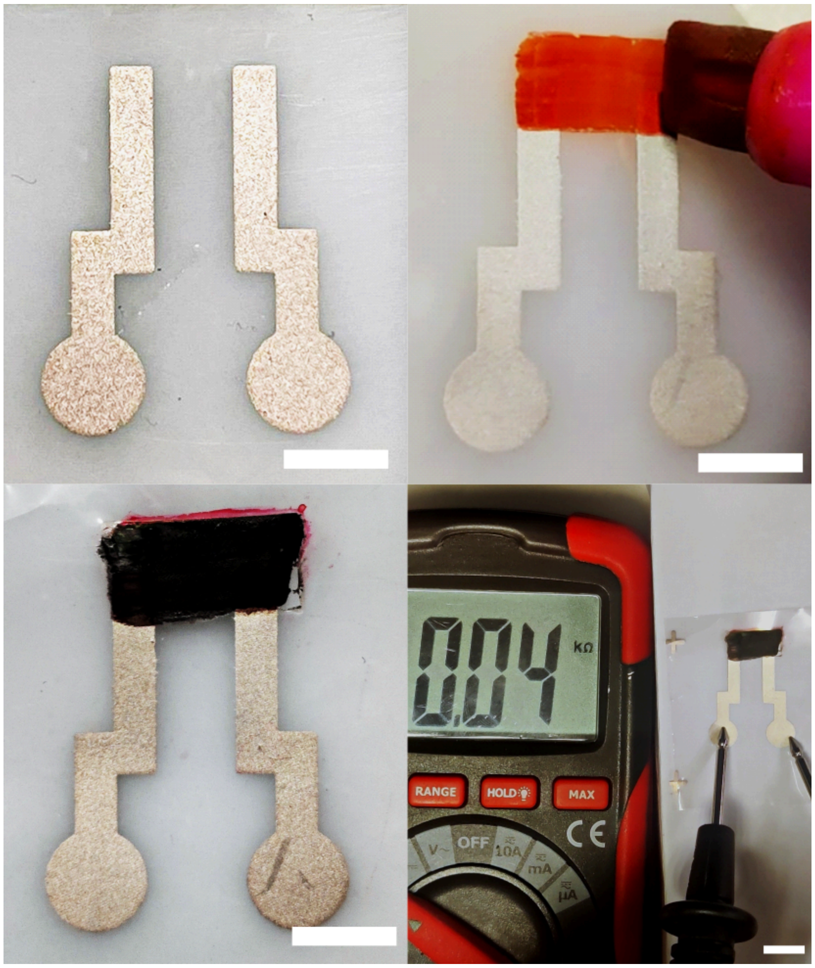

Figure S25: PPS approach for integration with printed and flexible electronics (scale bar = 5 mm)
